# Perinatal mortality and other severe adverse outcomes following planned birth at 39 weeks versus expectant management in low-risk women: a population based cohort study

**DOI:** 10.1016/j.eclinm.2025.103076

**Published:** 2025-01-25

**Authors:** Kylie Crawford, Waldemar A. Carlo, Anthony Odibo, Aris Papageorghiou, William Tarnow-Mordi, Sailesh Kumar

**Affiliations:** a Mater Research Institute - University of Queensland, Aubigny Place, Raymond Terrace, South Brisbane, Queensland, 4101, Australia; b School of Medicine, The University of Queensland, Herston, Queensland, 4006, Australia; c National Health and Medical Research Council Centre for Research Excellence in Stillbirth, South Brisbane, Queensland, 4101, Australia; d Division of Neonatology, Department of Pediatrics, University of Alabama at Birmingham, Birmingham, UK; e Division of Maternal-Fetal Medicine, Department of Obstetrics and Gynecology, University Health, Kansas City, Missouri, USA; f Nuffield Department of Women's & Reproductive Health, University of Oxford, Level 3, Women's Centre, John Radcliffe Hospital, Oxford, OX3 9DU, UK; g National Health and Medical Research Council Clinical Trials Centre, University of Sydney, Camperdown, Australia

**Keywords:** Pregnancy, Small for gestational age, Fetal growth restriction, Large for gestational age, Low risk women, Nulliparous, Multiparous, Induction of labour, Caesarean section

## Abstract

**Background:**

Planned birth by induction of labour in low-risk, nulliparous women at 39^+0^–39^+6^ weeks gestation is associated with fewer caesarean sections, adverse maternal and neonatal outcomes and perinatal deaths compared with expectant management. However, the consequences of scheduled caesarean section in these women at this gestation are unclear. We compared outcomes following planned birth at 39^+0^–39^+6^ weeks gestation (either by induction of labour or scheduled caesarean section) to expectant management.

**Methods:**

The population included low-risk, singleton pregnancies between 2000 and 2021 in Queensland, Australia. Study outcomes were perinatal mortality (antepartum or intrapartum stillbirth and neonatal death), severe neonatal neurological morbidity and non-neurological morbidity, severe maternal outcome, maternal-infant separation, perineal trauma, shoulder dystocia, and caesarean birth. Multivariable models were built to determine risks of adverse outcomes for planned birth compared to expectant management. Subgroup analyses according to parity and birthweight were also performed. We calculated the number of planned births required that were associated with one less adverse outcome.

**Findings:**

In 472,520 low-risk pregnancies, planned birth at 39^+0^–39^+6^ weeks occurred in 97,438 (20.6%) women, of whom 39,697 (40.7%) underwent induction of labour and 57,741 (59.3%) had scheduled caesarean delivery. Planned birth was associated with 52% lower odds of perinatal mortality (adjusted Odds Ratio (aOR) 0.48; 95% CI 0.30, 0.76, p = 0.002), 62% lower odds of antepartum stillbirth (aOR 0.38; 95% CI 0.15, 0.97, p = 0.04), and 84% lower odds of intrapartum stillbirth by (aOR 0.16; 95% CI 0.04, 0.66, p = 0.01). It was also associated with reduction in the odds of severe neurological morbidity (aOR 0.46; 95% CI 0.39, 0.53, p = 0.00004), severe non-neurological morbidity (aOR 0.65; 95% CI 0.62, 0.68, p = 0.00004), and severe maternal outcome (aOR 0.95; 95% CI 0.92, 0.99, p = 0.008) but not maternal-infant separation (aOR 1.04; 95% CI 1.00, 1.08, p = 0.08). The reduction in odds for perinatal mortality, severe neurological, and non-neurological morbidity was greatest for birth by scheduled caesarean section. Compared to expectant management, planned birth by induction of labour was associated with reduced odds of caesarean delivery (aOR 0.54; 95% CI 0.51, 0.58, p = 0.00004), severe perineal trauma (aOR 0.53; 95% CI 0.45, 0.63, p = 0.00004), and shoulder dystocia (aOR 0.73; 95% CI 0.64, 0.84, p = 0.00004). Planned delivery of 2278 (95% CI 1760, 3231) women is associated with a reduction in one case of perinatal death, however significantly lower numbers are required for the other outcomes.

**Interpretation:**

Planned birth at 39^+0^–39^+6^ weeks in low-risk women was associated with lower odds of perinatal mortality and other adverse outcomes. Reductions in odds of adverse outcome were greater following scheduled caesarean section than induction of labour. Compared to expectant management, induction of labour was associated with lower odds of severe perineal trauma, shoulder dystocia, and caesarean birth. These findings generate further hypotheses that need to be tested in adequately powered randomised controlled trials.

**Funding:**

This study was supported by funds from the 10.13039/501100000925National Health and Medical Research Council and 10.13039/100015471Mater Foundation.

**Research in context**

**Evidence before this study**

Regardless of mode of birth, infants born at early term gestations (37^+0^–38^+6^ weeks) are at higher risk of complications, both in the short (respiratory complications, admission to the neonatal intensive care unit, hypoxic ischaemic encephalopathy, and seizures) and longer term (poorer school performance, behavioural problems, and cerebral palsy). Rates of neonatal and maternal adverse outcomes are also higher when birth occurs at 40- and 41-weeks compared to 39 weeks' gestation. Although perinatal and some obstetric outcomes are better following induction of labour at 39 weeks' gestation, it is not known if planned birth by scheduled caesarean section at the same gestation has similar benefits. We searched PubMed for articles published from database inception to 26 September 2024 with search terms “induction of labour”, “caesarean section”, “perinatal mortality”, “neonatal morbidity”, “shoulder dystocia”, “low risk women”, “maternal mortality”, and “maternal morbidity” in various combinations with no language restrictions.

**Added value of this study**

We used data from an Australian cohort of 472,520 low-risk women who either underwent planned birth (by induction of labour or scheduled caesarean section) at 39^+0^–39^+6^ weeks or who had expectant management. We compared planned birth and expectant management to evaluate differences in clinically important perinatal, obstetric, and maternal outcomes. Sub analyses according to parity and birthweight were also undertaken. For each adverse perinatal and maternal outcome, we calculated absolute risk reductions, and the number needed to deliver that was associated with one less adverse outcome. We also performed multivariable regression analyses, adjusting for clinically relevant confounders and interactions, to determine adjusted odds ratios for each adverse outcome. These observational data provide new insights and hypotheses about the potential benefits and risks of planned birth in a clinically relevant, generalisable population.

**Implications of all the available evidence**

Overall, planned birth at 39^+0^–39^+6^ weeks in low-risk women was associated with reduced odds of perinatal mortality, severe neonatal neurological and non-neurological morbidity, and severe maternal outcome. Induction of labour was associated with reduction in odds of severe perineal trauma, shoulder dystocia, and caesarean birth. Reductions in the odds of perinatal mortality and neonatal morbidity were greater following scheduled caesarean delivery than induction of labour. However, despite its large size, as in any observational study, these findings may be subject to unmeasured confounding or selection bias. Randomised trials in different countries, health economic settings and populations are therefore required to establish the safety, efficacy, and acceptability of a policy of planned birth at 39 weeks in low-risk women.

## Introduction

Without a valid medical or obstetric indication, planned early term birth (37^+0^–38^+6^ weeks) should be avoided because infants born at these gestations are at higher risk of respiratory complications, admission to the neonatal intensive care unit, hypoxic ischaemic encephalopathy, and seizures^1^, ^2^, ^3^, ^4^, ^5^, ^6^, ^7^, ^8^ and are vulnerable to longer term neurodevelopmental issues.^9^ Conversely, rates of adverse neonatal and maternal outcomes are higher at 40- and 41-weeks' gestation compared to birth at 39 weeks.^10^^,^^11^ Thus, for women that reach 39^+0^ weeks, the question of whether to continue the pregnancy or to have a planned birth becomes pertinent.

Recent evidence suggests that, compared to expectant management, perinatal and some obstetric outcomes are better following planned birth at 39 weeks gestation.^12^^,^^13^ In the “A Randomized Trial of Induction Versus Expectant Management” (ARRIVE) study^14^ of 6106 low-risk, nulliparous women, planned induction of labour at 39 weeks resulted in a 20% reduction in the primary adverse perinatal composite outcome (4.3% vs. 5.4%; RR 0.80, 95% CI 0.64–1.00, p = 0.049), although this did not reach the threshold for statistical significance (set at 0.046). The frequency of caesarean delivery was 16% lower in the induction group (18.6% vs. 22.2%; RR 0.84, 95% CI 0.76–0.93, p < 0.001) and women who were induced also reported lower intrapartum median pain scores and had greater sense of control and satisfaction during labour. A subsequent desirability-of-outcome-ranking analysis of this study showed that maternal-infant dyadic outcomes were significantly better in the induction of labour cohort.^15^ Given evidence from this and other trials and cohort studies,^16^ the American College of Obstetricians and Gynaecologists (ACOG) and the Society for Maternal-Fetal Medicine (SMFM) advises that induction of labour at 39 weeks for low-risk nulliparous women is a reasonable option.^17^

However, any population-wide, real-world policy of offering planned birth at 39 weeks may also result, for some women, a preference for a scheduled caesarean section instead of induction of labour.^18^, ^19^, ^20^ It is not known if such planned birth by caesarean section at the same gestation results in similar maternal, infant, and obstetric benefits. Indeed, there is concern that caesarean section rates in many high-income settings are already too high and should be reduced not just from a health economic perspective but also because of adverse implications for a woman's future reproductive health.^21^, ^22^, ^23^ Although the World Health Organisation recommends that population-level caesarean section rates should not exceed 10–15%,^24^ subsequent studies suggest that the optimal rate associated with lower neonatal and maternal mortality is higher at ∼19 caesarean deliveries per 100 live births.^25^ However, these and other studies do not differentiate between scheduled and emergency caesarean deliveries making like-for-like comparisons problematic. Also, many studies perform cross-sectional comparisons and do not evaluate risks based on delivery at 39 weeks versus delivery thereafter.

The primary aim of this study was to investigate differences in clinically important perinatal and maternal outcomes in low-risk women following **any type of planned birth** (induction of labour or scheduled caesarean section) at 39^+0^–39^+6^ weeks compared to expectant management. We hypothesised that planned birth would be associated with fewer major adverse perinatal, obstetric, and maternal outcomes compared with expectant management. We also performed subgroup analyses according to parity and birthweight category.

## Methods

### Study population

We conducted a cohort study utilising the Queensland Perinatal Data Collection registry^26^ which contains deidentified maternal and perinatal information of all livebirths, stillbirths, and neonatal deaths between 2000 and 2021 in the state of Queensland, Australia. Institutional ethical approvals including waiver of participant consent were granted by the Metro North Hospital and Health Service Human Research Ethics Committee (Reference number: LNR/219/QRBC/53154). Our study population consisted of low-risk women with singleton pregnancies at or after 39^+0^ weeks. We defined low risk women as those without hypertension, diabetes mellitus, antepartum haemorrhage, maternal age <20 or ≥40 years, and body mass index ≥35 kg/m^2^. We also excluded women with infants with major structural abnormalities, or who had known genetic or chromosomal conditions and those that laboured spontaneously before 40^+0^ weeks. Smoking, illicit drug and alcohol use were not eliminated from our definition of low-risk pregnancies, but rather adjusted for in our analyses.

### Exposure

Planned birth was defined as induction of labour or scheduled caesarean section between 39^+0^ and 39^+6^ weeks gestational age. The comparison group was women who were expectantly managed—this group comprised women who continued their pregnancy to ≥40^+0^ weeks. We then examined planned birth as a categorical variable with 3 groups in multivariable logistic regression modelling to enable direct comparison of outcomes between expectant management, induction of labour, and scheduled caesarean section between 39^+0^ and 39^+6^ weeks.

### Outcomes

Study outcomes were: perinatal mortality, severe neonatal neurological morbidity, severe neonatal non-neurological morbidity, severe maternal outcome, maternal-infant separation, severe perineal trauma, shoulder dystocia, and caesarean birth.

Perinatal mortality was defined as antepartum stillbirth, intrapartum stillbirth, or neonatal mortality. Antepartum stillbirth was defined as foetal death in utero before the onset of labour, intrapartum stillbirth was defined as foetal death after commencement of labour but before birth, and neonatal mortality was defined as death within 28-days of birth. Severe neonatal neurological morbidity was a composite of neonatal encephalopathy, neonatal seizures, or intracerebral haemorrhage. Severe neonatal non-neurological morbidity was a composite of sepsis, necrotising enterocolitis, severe birth trauma, severe hypoglycaemia or prolonged nursery admission (defined as admission to the neonatal intensive care unit for ≥24 h or special care nursery for ≥7 days). Severe maternal outcome was a composite of maternal death, transfer to the intensive care unit, severe post-partum haemorrhage, post-partum sepsis, or postnatal length of stay ≥5 days. Maternal-infant separation was defined as neonatal nursery admission for ≥24 h. Severe perineal trauma was defined as third and fourth degree perineal tears. International Classification of Disease—10 (ICD-10) codes used to define clinical outcomes are listed in Supplementary Table S1.

All outcomes were analysed as binary variables and were mutually exclusive according to temporal sequence and severity. Thus, foetuses alive at the start of labour were not at risk of antepartum stillbirth, intrapartum stillbirths could not be classified as neonatal deaths, neonates who died could not be classified as having severe neurological morbidity or severe non-neurological morbidity. Shoulder dystocia, severe perineal trauma and caesarean section were only analysed for women attempting a vaginal birth because these consequences were not relevant in women undergoing elective caesarean section.

### Confounding and effect measure modification

We defined a potential confounder as a variable that is associated with both the exposure and the outcome but is not on the causal pathway between the exposure and the outcome. An effect measure modifier (or interaction) was defined as a variable that modifies the apparent effect of the exposure on the outcome but may not be associated with both the exposure and the outcome. We determined each type of variable based on clinical relevance—an approach consistent with recent methodological papers that have highlighted the importance of incorporating clinically important factors rather than using a purely data-driven model building approach.^27^^,^^28^ We determined that clinically relevant confounders of the association between planned birth at 39^+0^ and 39^+6^ weeks vs. expectant management and our study outcomes included maternal age, body mass index, country of birth, socioeconomic status, smoking, alcohol consumption, use of illicit drugs, previous history of stillbirth, assisted conception, and infant sex. Year of birth was also considered a confounder to reflect potential change in practice over the study period. Country of birth^29^ was used as a proxy for the confounding role of ethnicity because data on ethnicity were not available and it was considered important to incorporate a variable related to genetic drivers of health.^30^, ^31^, ^32^ Socioeconomic status was defined as deciles of the socioeconomic index for areas (SEIFA) score generated by the Australian Bureau of Statistics.^33^ This score ranks Australian geographical regions according to socioeconomic advantage based on information from the five-yearly census. Relative socioeconomic deprivation is defined as a SEIFA score in the lowest decile.^33^ Smoking, drug, and alcohol use were captured as a combination of self-reported and ICD-10 codes (Supplementary Table S1). Clinically relevant effect measure modifiers included parity (nulliparous vs. multiparous) and birthweight centile. Gestational age birthweight centiles were used to define infants that were small for gestational age (birthweight <10th centile), appropriate for gestational age (birthweight 10th–90th centile), and large for gestational age (birthweight >90th centile).^34^

### Missing data

Missing values were imputed using multivariate imputation using chained equations with two imputations (10 iterations) and the results of each imputation were combined using Rubin's rules.^35^ We considered that two imputations were sufficient because of the low percentages of missing data for most imputed variables and the consistency of results between imputations.^36^ Body mass index and birthweight centile were imputed using ordinal logistic regression, infant sex and country of birth using multinomial logistic regression, whilst socioeconomic deprivation, smoking, previous stillbirth and assisted conception were imputed using logistic regression. Imputations of missing values were performed with stillbirth, neonatal death, severe neurological morbidity, severe non-neurological morbidity, severe maternal outcome, and year of birth as covariates. Imputation of missing data was deemed appropriate after cross-tabulations revealed similar proportions of adverse perinatal outcomes in those with complete covariates compared to those in the imputed dataset.

### Statistical analyses

#### Incidence rates, absolute risk reductions and number needed to deliver

For antepartum stillbirth, we calculated and plotted Kaplan Meier estimates for the probability of antepartum stillbirth against gestational age at delivery. We then calculated the unadjusted incidence rate (number of adverse events divided by the number of pregnancies at risk) per 1000 births and 95% Confidence Intervals (95% CI) for all adverse outcomes. The absolute risk reductions (95% CI) were calculated by subtracting incidence rates for planned birth at 39^+0^–39^+6^ weeks from the incidence rates for expectant management. In this observational study, the number needed to deliver that was associated with one less adverse outcome (equivalent to the number needed to prevent one adverse outcome in a randomised controlled trial) was calculated as the reciprocal of the absolute risk reduction.

#### Multivariable logistic regression models

Preliminary screening of potential confounders was performed by calculating total numbers and percentages of births for all clinically relevant confounders. Univariable logistic regression models were built to provide unadjusted odds ratios (OR) and 95% CI of adverse outcomes for planned birth, compared to expectant management.

Interaction terms between planned birth and clinically relevant effect measure modifiers (parity and birthweight centiles) were then evaluated. Multivariable logistic regression models to provide adjusted ORs (aOR) and 95% CI were developed using forward selection of all clinically relevant confounders and significant effect measure modifiers (interactions), followed by stepwise backward elimination to avoid bias resulting from including inappropriate covariates.^27^^,^^28^^,^^37^ Continuous variables were categorised if linearity was absent in the log odds.^38^ Wald tests were used to evaluate significance using p < 0.05 for categorical variables and interaction terms.^39^ Robust standard errors^40^ (Huber-White Sandwich estimators) were used to account for clustering at the maternal level because subsequent pregnancies in the same woman are assigned new identification numbers in the Queensland Perinatal Dataset. Robust standard errors also protect against unknown clustering at the hospital level resulting from differences in obstetric or neonatal management practice. Models were built for all primary and secondary outcomes. Subgroup analyses were performed for outcomes that had significant interaction terms and sufficient events per variable.^28^ Models were repeated using planned birth as a 3-item categorical variable to enable direct comparison of the association of planned birth at 39^+0^–39^+6^ weeks versus induction of labour versus scheduled caesarean section or expectant management with adverse outcomes. To ascertain if exclusion of pregnancies resulting in spontaneous labour at 39^+0^–39^+6^ weeks (138,614 pregnancies) or those complicated by hypertension and/or diabetes mellitus and/or antepartum haemorrhage (85,942 pregnancies) influenced the study findings, sensitivity analyses were undertaken re-including these cohorts, respectively. Data were analysed using Stata 18.5® (Statacorp, College Station, TX, USA).

### Role of the funder

The funders had no role in analyses of the data, writing of the manuscript or the decision to submit.

## Results

**Fig. 1:**
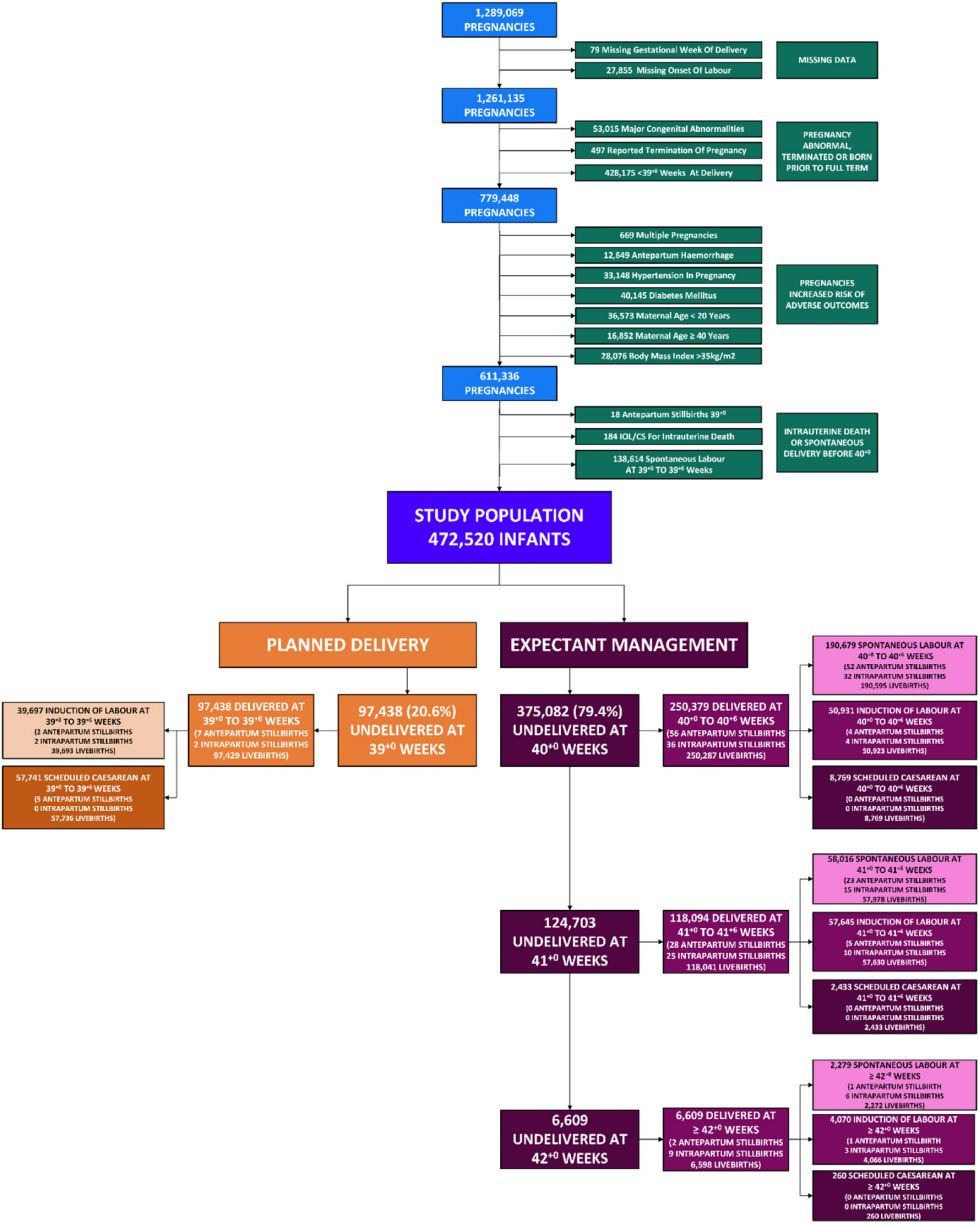
**Study flow chart**. Source Population: blue, Excluded pregnancies: green, Study population: purple, Planned Delivery at 39^+0^–39^+6^: orange, Expectant Management: magenta.

Of 1,289,069 births between 2000 and 2021, our final study population comprised of 472,520 low-risk singleton pregnancies ≥39^+0^ weeks (Fig. 1). There were no missing data for any of the study outcomes. Planned birth at 39^+0^–39^+6^ weeks occurred in 97,438 (20.6%) cases—of whom, 39,697 (40.7%) had induction of labour, and 57,741 (59.3%) had scheduled caesarean section. Parity and birthweight centile were significant effect measure modifiers of planned birth for many of the study outcomes (Supplementary Table S2). Table 1 presents the characteristics of the planned birth vs. expectant management cohorts and pregnancy outcomes. There were 281 cases of perinatal death (0.06% of the population) consisting of 93 (0.02%) antepartum stillbirths, 72 (0.02%) intrapartum stillbirths, and 116 (0.02%) neonatal deaths. Severe neonatal neurological morbidity occurred in 3566 (0.75%) infants and severe non-neurological morbidity occurred in 29,557 (6.3%) infants. Severe maternal outcome occurred in 49,680 (10.5%) pregnancies and there were 39,303 (8.3%) instances of maternal-infant separation. Of those attempting vaginal birth, there were 9473 (2.4%) cases of severe perineal trauma, 6341 (1.6%) cases of shoulder dystocia, and 71,177 (17.2%) caesarean births after intended vaginal delivery. The probability of an antepartum stillbirth increased with rising gestational age (Supplementary Figure S1).

**Table 1:** Population characteristics of women in the planned birth and expectant management cohorts.

	Total	Expectant management	IOL 39^+0^–39^+6^	Scheduled caesarean at 39^+0^–39^+6^	p-value
	N = 472,520	N = 375,082	N = 39,697	N = 57,741	
**Maternal age (years)**	29 (26, 33)	29 (25, 32)	30 (27, 33)	31 (28, 34)	<0.001
**Maternal BMI (kg/m**^**2**^**)**					<0.001
Underweight ≤18 kg	20,438 (4.3%)	15,721 (4.2%)	2181 (5.5%)	2536 (4.4%)	
Normal weight 19–24	161,161 (34.1%)	124,447 (33.2%)	15,960 (40.2%)	20,754 (35.9%)	
Overweight 25–29	79,351 (16.8%)	59,463 (15.9%)	7746 (19.5%)	12,142 (21.0%)	
Obese ≥30	37,077 (7.8%)	27,111 (7.2%)	3774 (9.5%)	6192 (10.7%)	
Missing	174,493 (36.9%)	148,340 (39.5%)	10,036 (25.3%)	16,117 (27.9%)	
**Mother's country of birth**					<0.001
USA, Can, Aus, NZ, & Europe	398,716 (84.4%)	316,883 (84.5%)	33,469 (84.3%)	48,364 (83.8%)	
Indigenous Australian	23,692 (5.0%)	19,594 (5.2%)	1766 (4.4%)	2332 (4.0%)	
Latin America & Caribbean	2800 (0.6%)	2023 (0.5%)	315 (0.8%)	462 (0.8%)	
NE, SE Asia & Oceana (excluding ANZ)	26,927 (5.7%)	21,105 (5.6%)	2240 (5.6%)	3582 (6.2%)	
South and Central Asia	8960 (1.9%)	6630 (1.8%)	1009 (2.5%)	1321 (2.3%)	
Sub-Saharan Africa	7391 (1.6%)	5649 (1.5%)	603 (1.5%)	1139 (2.0%)	
West Asia, North Africa & Middle East	4022 (0.9%)	3186 (0.8%)	295 (0.7%)	541 (0.9%)	
Missing	12 (0.0%)	12 (0.0%)	0 (0.0%)	0 (0.0%)	
**Low socioeconomic status**^a^	41,032 (8.7%)	34,086 (9.1%)	2759 (7.0%)	4187 (7.3%)	<0.001
Missing	4118 (0.9%)	3014 (0.8%)	467 (1.2%)	637 (1.1%)	
**Smoking in pregnancy**	45,628 (9.7%)	37,015 (9.9%)	3486 (8.8%)	5127 (8.9%)	<0.001
Missing	120,224 (25.4%)	103,272 (27.5%)	6854 (17.3%)	10,098 (17.5%)	
**Alcohol in pregnancy**^d^	760 (0.2%)	661 (0.2%)	53 (0.1%)	46 (0.1%)	<0.001
**Illicit drugs in pregnancy**^d^	2372 (0.5%)	1862 (0.5%)	253 (0.6%)	257 (0.4%)	<0.001
**Previous stillbirth**	4410 (0.9%)	3014 (0.8%)	698 (1.8%)	698 (1.2%)	<0.001
Missing	4 (0.0%)	4 (0.0%)	0 (0.0%)	0 (0.0%)	
**Assisted conception**	15,411 (3.3%)	10,043 (2.7%)	2575 (6.5%)	2793 (4.8%)	<0.001
Missing	11 (0.0%)	10 (0.0%)	0 (0.0%)	1 (0.0%)	
**Infant weight (g)**	3609 ± 437	3639 ± 433	3465 ± 439	3511 ± 423	<0.001
**Infant sex**					0.85
Male	239,330 (50.6%)	190,066 (50.7%)	20,036 (50.5%)	29,228 (50.6%)	
Female	233,187 (49.3%)	185,013 (49.3%)	19,661 (49.5%)	28,513 (49.4%)	
Missing	3 (0.0%)	3 (0.0%)	0 (0.0%)	0 (0.0%)	
**Gestational age in days**	280 (280, 287)	282 (280, 287)	274 (273, 276)	273 (273, 275)	<0.001
**Method of birth**					<0.001
Vaginal non-instrumental	290,337 (61.4%)	262,698 (70.0%)	27,639 (69.6%)		
Forceps	13,786 (2.9%)	12,419 (3.3%)	1367 (3.4%)		
Vacuum	39,479 (8.4%)	35,073 (9.4%)	4406 (11.1%)		
Scheduled caesarean	69,203 (14.6%)	11,462 (3.1%)		57,741 (100.0%)	
Emergency caesarean	59,715 (12.6%)	53,430 (14.2%)	6285 (15.8%)		
**Perinatal mortality**	281 (0.1%)	257 (0.1%)	12 (0.0%)	12 (0.0%)	<0.001
**Stillbirth**	165 (0.0%)	156 (0.0%)	4 (0.0%)	5 (0.0%)	<0.001
Antepartum	93 (0.0%)	86 (0.0%)	2 (0.0%)	5 (0.0%)	0.007
Intrapartum	72 (0.0%)	70 (0.0%)	2 (0.0%)	0 (0.0%)	<0.001
**Neonatal death**	116 (0.0%)	101 (0.0%)	8 (0.0%)	7 (0.0%)	0.072
**Severe neurological morbidity**^b^	3566 (0.8%)	3130 (0.9%)	287 (0.8%)	149 (0.3%)	<0.001
All birth asphyxia	3611 (0.8%)	3173 (0.8%)	288 (0.7%)	150 (0.3%)	<0.001
HIE	263 (0.1%)	245 (0.1%)	16 (0.0%)	2 (0.0%)	<0.001
Seizures	341 (0.1%)	301 (0.1%)	19 (0.0%)	21 (0.0%)	<0.001
Intracerebral haemorrhage	37 (0.0%)	34 (0.0%)	1 (0.0%)	2 (0.0%)	0.17
**Non-neurological morbidity**^b^	29,557 (6.3%)	24,748 (6.7%)	2889 (7.3%)	1920 (3.3%)	<0.001
Sepsis	21,811 (4.6%)	18,979 (5.1%)	1996 (5.0%)	836 (1.4%)	<0.001
Necrotising enterocolitis	7 (0.0%)	6 (0.0%)	0 (0.0%)	1 (0.0%)	0.72
Birth trauma	3922 (0.8%)	3416 (0.9%)	377 (0.9%)	129 (0.2%)	<0.001
Hypoglycaemia	6460 (1.4%)	4762 (1.3%)	808 (2.0%)	890 (1.5%)	<0.001
**Maternal-infant separation**	39,303 (8.3%)	32,030 (8.5%)	3389 (8.5%)	3884 (6.7%)	<0.001
Admission to NICU	2692 (0.6%)	2198 (0.6%)	225 (0.6%)	269 (0.5%)	0.002
Admission to SCN	37,454 (7.9%)	30,514 (8.2%)	3237 (8.2%)	3703 (6.4%)	<0.001
**Severe adverse maternal outcome**^b^	49,680 (10.5%)	40,233 (10.7%)	4206 (10.6%)	5241 (9.1%)	<0.001
Maternal transfer	3781 (0.8%)	3222 (0.9%)	173 (0.4%)	386 (0.7%)	
Maternal death	4 (0.0%)	4 (0.0%)	0 (0.0%)	0 (0.0%)	
Missing	1316 (0.3%)	1316 (0.4%)	0 (0.0%)	0 (0.0%)	
Post-partum haemorrhage	30,672 (6.5%)	25,871 (6.9%)	2788 (7.0%)	2013 (3.5%)	<0.001
Post-partum sepsis	4538 (1.0%)	3701 (1.0%)	392 (1.0%)	445 (0.8%)	<0.001
Maternal stay >5 days	13,559 (2.9%)	9827 (2.6%)	1065 (2.7%)	2667 (4.6%)	<0.001
**Grade 3 or 4 perineal trauma**	9473 (2.3%)	8871 (2.4%)	602 (1.5%)		<0.001
**Shoulder dystocia**	6341 (1.6%)	5778 (1.6%)	563 (1.4%)		0.009
**Caesarean birth**^c^	71,177 (17.2%)	64,892 (17.3%)	6285 (15.8%)		

Legend: Values represent Number (column percentages), median(25%,75%) or mean ± standard deviation; GA Gestational Age; BMI Body mass Index (kg/m^2^); USA United States of America; Can Canada; Aus Australia; NZ New Zealand; ANZ Australia & New Zealand; NE North-East, SE South-East.

a Socioeconomic status is lowest decile of socioeconomic index for areas (SEIFA) score generated by the Australian Bureau of Statistics.

b Note individuals may have multiple components of composite outcomes, thus components will not equate to total.

c After attempted vaginal delivery, thus in planned birth this represents caesarean birth after IOL.

d Captured from ICD-10 codes.

**Fig. 2:**
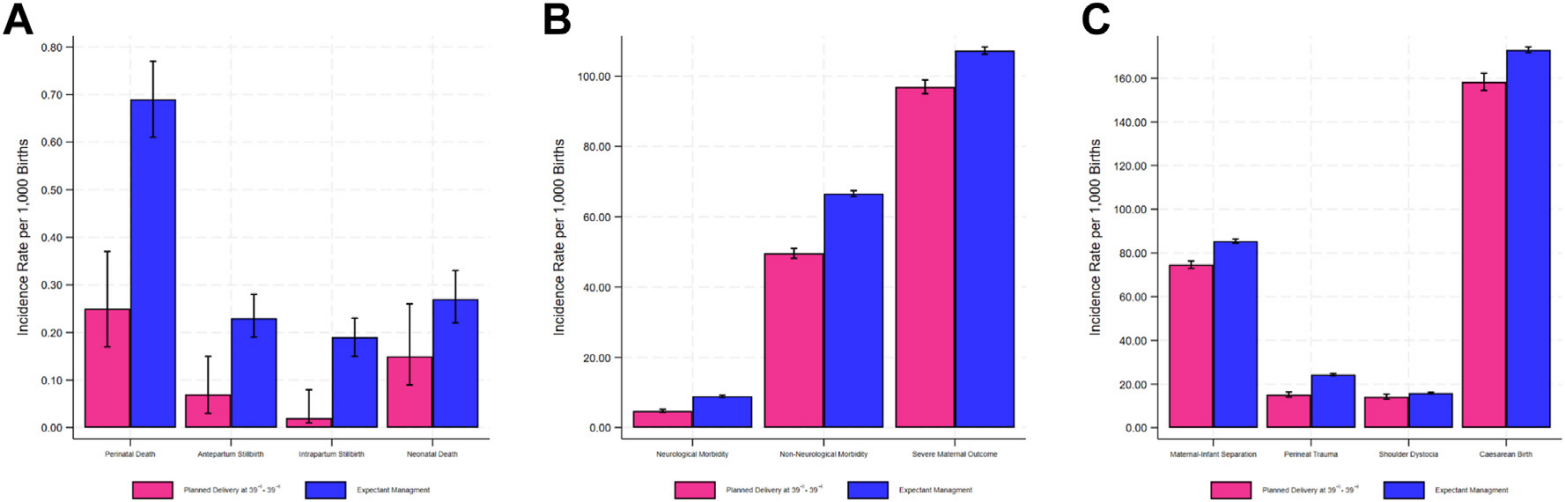
**Incidence rates and 95% confidence intervals for adverse perinatal, maternal and infant outcomes for planned birth at 39**^**+0**^**–39**^**+6**^**vs. expectant management**. A: Perinatal death, antepartum stillbirth, intrapartum stillbirth, and neonatal death; B: Severe Neurological Morbidity, Severe Non-Neurological morbidity, and Severe Maternal Outcome; C: Maternal-infant Separation, Severe Perineal Trauma, Shoulder Dystocia, and Caesarean birth following intended vaginal delivery.

**Fig. 3:**
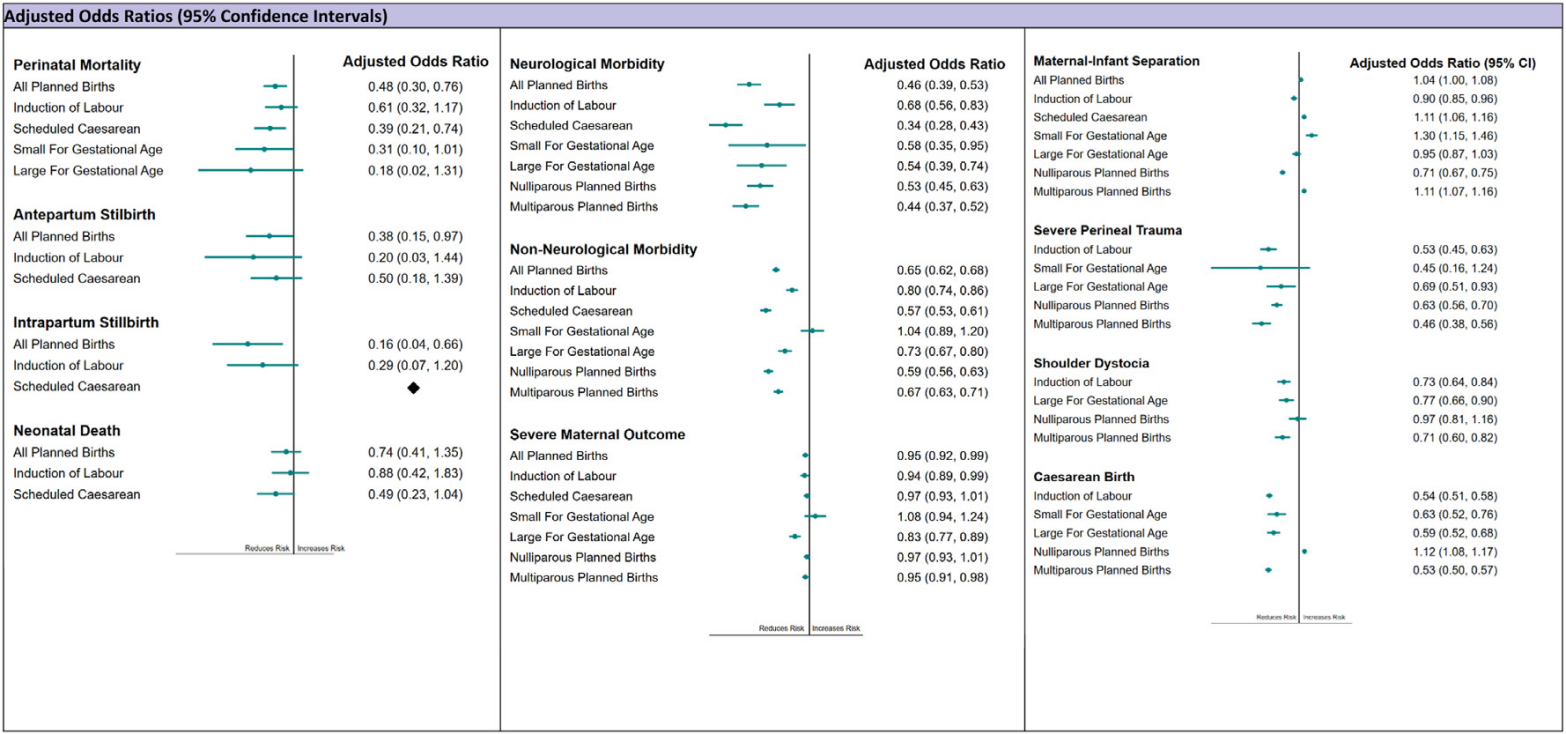
**Adjusted odds ratios from multivariable logistic regression models of planned birth at 39**^**+0**^**–39**^**+6**^**weeks compared to expectant management for study outcomes**. Perinatal Mortality: Antepartum Stillbirth, Intrapartum Stillbirth, Neonatal Death; Severe Perineal Trauma: Grades 3 or 4 Perineal Tears, Caesarean Birth: Caesarean birth following intended vaginal delivery; ♦No Intrapartum stillbirths observed with elective caesarean section.

Incidence rates per 1000 births for the various study outcomes for the planned and expectant management cohorts are shown in Fig. 2. Compared to expectant management, planned birth at 39^+0^–39^+6^ weeks was associated with lower odds of overall perinatal mortality (aOR 0.48; 95% CI 0.30, 0.76, p = 0.002), severe neonatal neurological mortality (aOR 0.46; 95% CI 0.39, 0.53, p = 0.00004), severe neonatal non-neurological morbidity (aOR 0.65; 95% CI 0.62, 0.68, p = 0.00004), and severe maternal outcome (aOR 0.95; 95% CI 0.92, 0.99, p = 0.008), but not maternal-infant separation (aOR 1.04; 95% CI 1.00, 1.08, p = 0.075). Induction of labour at 39^+0^–39^+6^ weeks was associated with lower odds of severe perineal trauma (aOR 0.53; 95% CI 0.45, 0.63, p = 0.00004), shoulder dystocia (aOR 0.73; 95% CI 0.64, 0.84, p = 0.00004), and caesarean birth (aOR 0.54; 95% CI 0.51, 0.58, p = 0.00004) (Fig. 3, Supplementary Tables S3–S5).

In sensitivity analyses including pregnancies that resulted in spontaneous labour at 39^+0^–39^+6^ weeks, the point estimates for the various study outcomes and sub-groups were very similar as in the primary analyses (Supplementary Figure S2). In further sensitivity analyses including pregnancies with hypertension, diabetes mellitus or antepartum haemorrhage, planned birth was again associated with reduction in odds of perinatal mortality, severe neurological and non-neurological morbidity, severe perineal trauma, shoulder dystocia, and caesarean birth. The odds of severe maternal outcome and maternal infant separation were however increased (Supplementary Figure S3).

The association between overall planned birth and study outcomes was then examined within cohorts according to birthweight centile and parity. For severe neonatal neurological morbidity, planned birth was associated with lower odds in the following cohorts: small for gestational age (0.58, 95% CI 0.35, 0.95) and large for gestational age infants (0.54, 95% CI 0.39, 0.74), nulliparous (0.53, 95% CI 0.45, 0.63), and multiparous women (0.44, 95% CI 0.37, 0.52). For severe neonatal non-neurological morbidity, planned birth was associated with reduction in odds for nulliparous (0.59, 95% CI 0.56, 0.63) and multiparous women (0.67, 95% CI 0.63, 0.71), and those with a large for gestational age infant (0.73, 95% CI 0.67, 0.80). Planned birth was associated with increased odds for maternal-infant separation in multiparous women (1.11, 95% CI 1.06, 1.16) and those with small for gestational age infants (1.30, 95% CI 1.15, 1.46), but reduced odds in nulliparous women (0.71, 95% CI 0.67, 0.75). Induction of labour was associated with reduced odds of severe perineal trauma in nulliparous (0.63, 95% CI 0.56, 0.70), multiparous (0.46, 95% CI 0.38, 0.56) and women with large for gestational age infants (0.69, 95% CI 0.51, 0.93). Induction of labour was also associated with reduced odds of shoulder dystocia in multiparous women (0.71, 95% CI 0.60, 0.82) and those with large for gestational age infants (0.77, 95% CI 0.66, 0.90). In nulliparous women, induction of labour compared to expectant management was associated with increased odds of caesarean section (1.12, 95% CI 1.08, 1.17).

We then evaluated the associations between method of planned birth (induction of labour or scheduled caesarean) and study outcomes (Table 2, Table 3). Compared to expectant management, planned birth by caesarean section was associated with 61% lower odds of perinatal mortality (aOR 0.39; 95% CI 0.21, 0.74), but planned birth by induction of labour was not (aOR 0.61; 95% CI 0.32, 1.17). Similarly, for severe neonatal neurological morbidity and non-neurological morbidity, scheduled caesarean section was also associated with a greater reduction in odds for both outcomes. However, scheduled caesarean birth was associated with higher odds (aOR 1.11; 95% CI 1.06, 1.16) of maternal-infant separation whilst induction of labour was associated with lower odds (aOR 0.90; 95% CI 0.85, 0.96) of this outcome. Induction of labour was associated with reduced odds of severe maternal outcome, (aOR 0.94; 95% CI 0.89, 0.99) but scheduled caesarean was not (aOR 0.97; 95% CI 0.93, 1.01).

**Table 2:** Multivariable logistic regression analyses of the effect of method of planned birth at 39^+0^–39^+6^ weeks compared to expectant management on perinatal mortality, severe neurological morbidity and severe non-neurological morbidity for all births, small for gestational age, large for gestational age, nulliparous and multiparous cohorts.

	Perinatal mortality				Severe neurological morbidity				Severe non-neurological morbidity			
	OR (95% CI)	p-value	aOR (95% CI)	p-value	OR (95% CI)	p-value	aOR (95% CI)	p-value	OR (95% CI)	p-value	aOR (95% CI)	p-value
**All births N = 472,520**												
Induction of labour N = 39,697	0.44 (0.25, 0.79)	0.006	0.61 (0.32, 1.17)^a^	0.14	0.87 (0.77, 0.98)	0.03	0.68 (0.56, 0.83)^c^	0.0001	1.11 (1.07, 1.15)	<0.0001	0.80 (0.74, 0.86)^h^	<0.0001
Scheduled caesarean N = 57,741	0.30 (0.17, 0.54)	0.0001	0.39 (0.21, 0.74)^a^	0.004	0.30 (0.25, 0.35)	<0.0001	0.34 (0.28, 0.43)^c^	<0.0001	0.48 (0.46, 0.51)	<0.0001	0.57 (0.53, 0.61)^h^	<0.0001
**Small for gestational age births N = 37,623**												
Induction of labour N = 3460	0.17 (0.02, 1.22)	0.08	0.21 (0.03, 1.51)^b^	0.12	0.8 (0.56, 1.15)	0.23	0.71 (0.37, 1.37)^d^	0.31	1.58 (1.42, 1.76)	<0.0001	1.26 (1.03, 1.54)^i^	0.02
Scheduled caesarean N = 3178	0.37 (0.09, 1.51)	0.17	0.42 (0.10, 1.73)^b^	0.23	0.25 (0.13, 0.47)	<0.0001	0.48 (0.24, 0.95)^d^	0.04	0.71 (0.61, 0.82)	<0.0001	0.88 (0.72, 1.07)^i^	0.19
**Large for gestational age births N = 52,560**												
Induction of labour N = 4720	##	##	##	##	1.18 (0.88, 1.58)	0.27	1.00 (0.68, 1.46)^e^	0.98	1.06 (0.96, 1.17)	0.28	0.76 (0.65, 0.87)^i^	0.0001
Scheduled caesarean N = 7598	##	##	##	##	0.32 (0.21, 0.48)	<0.0001	0.29 (0.18, 0.47)^e^	<0.0001	0.69 (0.63, 0.76)	<0.0001	0.72 (0.65, 0.81)^i^	<0.0001
**Nulliparous N = 195,351**												
Induction of labour N = 16,119	NA	NA	NA	NA	0.90 (0.77, 1.06)	0.20	0.70 (0.58, 0.84)^f^	0.0002	1.22 (1.16, 1.29)	<0.0001	1.23 (1.07, 1.42)^j^	0.004
Scheduled caesarean N = 12,250	NA	NA	NA	NA	0.29 (0.21, 0.39)	<0.0001	0.29 (0.21, 0.41)^f^	<0.0001	0.32 (0.29, 0.35)	<0.0001	0.52 (0.39, 0.69)^j^	<0.0001
**Multiparous N = 277,167**												
Induction of labour N = 23,578	NA	NA	NA	NA	0.9 (0.75, 1.08)	0.25	0.65 (0.52, 0.81)^g^	0.0002	1.05 (0.98, 1.12)	0.18	1.27 (1.04, 1.56)^k^	0.02
Scheduled caesarean N = 45,491	NA	NA	NA	NA	0.39 (0.32, 0.47)	<0.0001	0.33 (0.26, 0.42)^g^	<0.0001	0.77 (0.73, 0.81)	<0.0001	0.88 (0.73, 1.07)^k^	0.22

Legend: OR (95% CI) Odds Ratio and 95% Confidence Interval; aOR Adjusted Odds Ratio; NA not applicable because interaction not significant; ## analyses not performed because insufficient failures (≤10) per variable.

a Adjusted for BMI, smoking, illicit drugs, previous stillbirth and year of birth, interaction planned birth#birthweight centile.

b Adjusted for previous stillbirth, infant sex, and year of birth.

c Adjusted for maternal age, BMI, maternal country of birth, low socioeconomic status, smoking, alcohol, illicit drugs, previous stillbirth, assisted conception, infant sex and year of birth, interactions planned birth#parity, and planned birth#birthweight centile.

d Adjusted for BMI, low socioeconomic status, smoking, illicit drugs, infant sex and year of birth, interaction planned birth#parity.

e Adjusted for BMI, smoking, assisted conception, infant sex and year of birth, interaction planned birth#parity.

f Adjusted for maternal age, BMI, low socioeconomic status, smoking, illicit drugs, assisted conception, infant sex and year of birth, interaction planned birth#birthweight centile.

g Adjusted for BMI, low socioeconomic status, smoking, alcohol, illicit drugs, previous stillbirth, infant sex and year of birth, interaction planned birth#birthweight centile.

h Adjusted for BMI, maternal country of birth, smoking, illicit drugs, assisted conception, infant sex, year of birth and interactions planned birth#parity and planned birth#birthweight centile.

i Adjusted for for BMI, maternal country of birth, smoking, illicit drugs, assisted conception, infant sex, year of birth and interactions planned birth#parity.

j Adjusted for for BMI, maternal country of birth, smoking, illicit drugs, assisted conception, infant sex and year of birth and interaction planned birth#birthweight centile.

k Adjusted for for BMI, maternal country of birth, smoking, illicit drugs, infant sex, year of birth.

**Table 3:** Multivariable logistic regression analyses of the association of method of planned birth at 39^+0^–39^+6^ weeks compared to expectant management with antepartum stillbirth, intrapartum stillbirth, neonatal death, maternal-infant separation and severe maternal outcome for all births, small for gestational age, large for gestational age, nulliparous and multiparous cohorts.

	Antepartum stillbirth				Intrapartum stillbirth				Neonatal death				Maternal-infant separation				Severe maternal outcome			
	OR (95% CI)	p value	aOR (95% CI)	p value	OR (95% CI)	p value	aOR (95% CI)	p value	OR (95% CI)	p value	aOR (95% CI)	p value	OR (95% CI)	p value	aOR (95% CI)	p-value	OR (95% CI)	p-value	aOR (95% CI)	p value
**All births N = 472,520**																				
Induction of labour N = 39,697	0.22 (0.05, 0.89)	0.03	0.20 (0.03, 1.44)^b^	0.11	0.27 (0.07, 1.11)	0.07	0.29 (0.07, 1.20)^c^	0.09	0.75 (0.37, 1.55)	0.44	0.88 (0.42, 1.83)^d^	0.73	1.00 (0.96, 1.04)	0.97	0.90 (0.85, 0.96)^e^	0.002	0.99 (0.95, 1.02)	0.42	0.94 (0.89, 0.99)^j^	0.02
Scheduled caesarean N = 57,741	0.38 (0.15, 0.93)	0.03	0.50 (0.18, 1.39)^b^	0.18	##	##	##	##	0.43 (0.20, 0.93)	0.03	0.49 (0.23, 1.04)^d^	0.07	0.77 (0.75, 0.80)	<0.0001	1.11 (1.06, 1.16)^e^	<0.0001	0.83 (0.81, 0.86)	<0.0001	0.97 (0.93, 1.01)^j^	0.09
**Small for gestational age births N = 37,623**																				
Induction of labour N = 3460	##	##	##	##	##	##	##	##	##	##	##	##	1.47 (1.33, 1.62)	<0.0001	1.39 (1.18, 1.63)^f^	0.0001	1.11 (0.99, 1.25)	0.08	0.99 (0.81, 1.22)^k^	0.93
Scheduled caesarean N = 3178	##	##	##	##	##	##	##	##	##	##	##	##	0.94 (0.84, 1.06)	0.32	1.24 (1.07, 1.44)^f^	0.004	1.00 (0.88, 1.14)	0.98	1.14 (0.96, 1.35)^k^	0.14
**Large for gestational age births N = 52,560**																				
Induction of labour N = 4720	##	##	##	##	##	##	##	##	##	##	##	##	0.88 (0.80, 0.97)	0.01	0.79 (0.69, 0.90)^g^	0.0005	0.97 (0.89, 1.06)	0.54	0.99 (0.88, 1.10)^l^	0.83
Scheduled caesarean N = 7598	##	##	##	##	##	##	##	##	##	##	##	##	0.84 (0.77, 0.91)	<0.0001	1.03 (0.94, 1.13)^g^	0.51	0.66 (0.61, 0.71)	<0.0001	0.75 (0.68, 0.82)^l^	<0.0001
**Nulliparous N = 195,351**																				
Induction of labour N = 16,119	NA	NA	NA	NA	NA	NA	NA	NA	##	##	##	##	1.07 (1.02, 1.12)	0.008	0.91 (0.86, 0.97)^h^	0.003	1.05 (1.00, 1.10)	0.04	1.04 (0.98, 1.10)^m^	0.17
Scheduled caesarean N = 12,250	NA	NA	NA	NA	NA	NA	NA	NA	##	##	##	##	0.50 (0.47, 0.54)	<0.0001	0.45 (0.41, 0.49)^h^	<0.0001	0.92 (0.87, 0.97)	0.002	0.88 (0.83, 0.94)^m^	0.0001
**Multiparous**^a^**N = 277,167**																				
Induction of labour N = 23,578	NA	NA	NA	NA	NA	NA	NA	NA	##	##	##	##	0.98 (0.93, 1.04)	0.53	0.94 (0.88, 1.01)^i^	0.10	0.96 (0.92, 1.01)	0.12	0.92 (0.87, 0.97)^n^	0.004
Scheduled caesarean^a^ N = 45,491	NA	NA	NA	NA	NA	NA	NA	NA	##	##	##	##	1.17 (1.13, 1.22)	<0.0001	1.20 (1.15, 1.26)^i^	<0.0001	0.95 (0.92, 0.99)	0.008	0.96 (0.92, 1.00)^n^	0.05

Legend: OR (95% CI) Odds Ratio and 95% Confidence Interval; aOR Adjusted Odds Ratio; NA not applicable because interaction not significant; ## analyses not performed because insufficient failures (≤10) per variable.

a When multiparous women without a previous caesarean section were excluded, the odds of maternal-infant separation remained significantly increased (aOR 1.40; 95% CI 1.28, 1.54).

b Adjusted for smoking and year of birth, interaction planned birth#birthweight centile.

c Adjusted for maternal age, previous stillbirth, and year of birth.

d Adjusted for illicit drugs and year of birth.

e Adjusted for maternal age, BMI, maternal country of birth, smoking, illicit drugs, previous stillbirth, assisted conception, infant sex and year of birth, interactions planned birth#parity, planned birth#birthweight centile.

f Adjusted for BMI, maternal country of birth, smoking, illicit drugs, assisted conception, infant sex, year of birth and interaction planned birth#parity.

g Adjusted for maternal age, BMI, maternal country of birth, smoking, illicit drugs, assisted conception, infant sex, year of birth and interaction planned birth#parity.

h Adjusted for maternal age, BMI, maternal country of birth, smoking, illicit drugs, assisted conception, infant sex, year of birth and interaction planned birth#birthweight centile.

i Adjusted for maternal age, BMI, maternal country of birth, smoking, illicit drugs, previous stillbirth, assisted conception, infant sex, year of birth and interaction planned birth#birthweight centile.

j Adjusted for maternal age, BMI, maternal country of birth, low SES, illicit drugs, previous stillbirth, assisted conception, interactions planned birth#birthweight centile and planned birth#parity.

k Adjusted for maternal age, maternal country of birth, low SES. Illicit drugs, previous stillbirth, assisted conception, year of birth and interaction planned birth#parity.

l Adjusted for maternal age, BMI, maternal country of birth. Illicit drugs, previous stillbirth, assisted conception, year of birth and interaction planned birth#parity.

m Adjusted for maternal age, BMI, maternal country of birth, illicit drugs, assisted conception, year of birth, interaction planned birth and birthweight centile.

n Adjusted for maternal age, BMI, maternal country of birth, low SES, smoking, illicit drugs, previous stillbirth, assisted conception, year of birth, interaction planned birth and birthweight centile.

Subgroup analyses by parity and birthweight (Table 2, Table 3), showed that compared to induction of labour, scheduled caesarean was associated with greater reduction in odds of severe neonatal neurological morbidity for nulliparous and multiparous women and those with either small or large for gestational age infants. For severe neonatal non-neurological morbidity, scheduled caesarean section compared to induction of labour was associated with lower odds in nulliparous women and women with large for gestational age infants. However, in nulliparous and multiparous women, induction of labour, was associated with increased odds of this outcome (23% and 27% respectively). For maternal infant separation, scheduled caesarean section was associated with 11% increased odds whilst induction of labour was associated with 10% lower odds of this outcome. For women with small for gestational age infants, both induction and scheduled caesarean section were associated with increased odds of maternal-infant separation whilst for nulliparous women, both methods of planned birth were associated with lower odds. In contrast, for multiparous women, scheduled caesarean section was associated with a 20% increase in the odds of this outcome. The increase in odds persisted even when multiparous women without a previous caesarean birth were excluded from the analysis (Table 3).

In Table 4 the absolute risk reductions per 1000 births and their reciprocals, the number needed to treat to ‘prevent’ one adverse perinatal or maternal outcome, i.e., associated with one less case, is presented for all planned births stratified by induction of labour and scheduled caesarean section. The number of planned births associated with one less case of perinatal mortality is 2278 (95% CI 1760, 3231). Substantially lower numbers of planned births are associated with one less case of severe neonatal neurological morbidity (236, 95% CI 209, 271), severe neonatal non-neurological morbidity (59, 95% CI 54, 65), severe maternal outcome (97, 95% CI 80, 124), and maternal-infant separation (93, 95% CI 79, 113). Broadly similar numbers were associated with one less case of severe perineal trauma (108, 95% CI 95, 126) and caesarean section (68, 95% CI 53, 95). Only 48 (95% CI 45, 52) planned births are associated with one less case of the combined outcome of perinatal mortality or severe neurological or non-neurological morbidity.

**Table 4:** Number needed to deliver at 39^+0^–39^+6^ weeks that is associated with one less adverse perinatal or maternal outcome or caesarean section.

Outcome	Number needed to deliver (95% CI)
**Perinatal mortality**^d^	
All planned births	2278 (1760, 3231)
Induction of labour	2612 (1744, 5198)
Scheduled caesarean section	2095 (1608, 3003)
**Antepartum stillbirth**	
All planned births	6353 (4359, 11,696)
Induction of labour	5590 (3789, 10,650)
Scheduled caesarean section	7008 (4297, 19,011)
**Intrapartum stillbirth**	
All planned births	6020 (4581, 8772)
Induction of labour	7337 (4572, 18,553)
Scheduled caesarean section	a
**Neonatal death**	
All planned births	8666 (4776, 46,512)
Induction of labour	b
Scheduled caesarean section	6752 (3965, 22,676)
**Severe neonatal neurological morbidity**^d^	
All planned births	236 (209, 271)
Induction of labour	876 (477, 5379)
Scheduled caesarean section	159 (147, 174)
**Severe neonatal non-neurological morbidity**^d^	
All planned births	59 (54, 65)
Induction of labour	c
Scheduled caesarean section	30 (29, 32)
**Severe maternal outcome**	
All planned births	97 (80, 124)
Induction of labour	b
Scheduled caesarean section	61 (52, 72)
**Maternal-infant separation**	
All planned births	93 (79, 113)
Induction of labour	b
Scheduled caesarean section	55 (49, 63)
**Severe perineal trauma**	
Induction of labour	108 (95, 126)
**Shoulder dystocia**	
Induction of labour	586 (339, 2143)
**Caesarean birth**^e^	
Induction of labour	68 (53, 95)

Legend: 95% CI 95% Confidence Interval.

a No intrapartum stillbirths with scheduled caesarean delivery.

b Values not statistically significant.

c Values increase rather than reduce the risk of adverse outcome.

d 48 (95% CI 45, 52) planned births are associated with one less case of perinatal mortality or severe neurological or non-neurological morbidity.

e Caesarean birth after intended vaginal delivery.

## Discussion

This population-based cohort study of low-risk women demonstrates that planned birth by induction of labour or elective caesarean section at 39^+0^–39^+6^ weeks compared to expectant management was associated with significantly reduced odds of perinatal mortality, severe neonatal morbidity (neurological and non-neurological), severe maternal outcome, severe perineal trauma, shoulder dystocia, and caesarean birth. Although planned birth overall was not associated with a difference in odds of maternal-infant separation after birth, induction of labour at 39^+0^–39^+6^ weeks was associated with 10% reduction of this outcome. For many of the outcomes investigated, scheduled caesarean section was more strongly associated with reduced odds of adverse outcome than induction of labour, both in the overall study population and in various subgroups.

The association of planned birth with adverse outcome in various subgroups (women with small for gestational age or large for gestational age infants, nulliparous, and multiparous) was variable and nuanced. Planned birth regardless of method, was associated with significantly reduced odds of severe neonatal neurological morbidity in women with small and large for gestational age infants as well as nulliparous and multiparous women. Planned birth was not associated with severe neonatal non neurological morbidity in small for gestational age infants but for those in the large for gestational age, nulliparous, and multiparous cohorts it was associated with lower odds of this outcome. For severe maternal outcome, planned birth was associated with 17% reduction in odds in the large for gestational age cohort compared to only 5% in multiparous women.

Many population-based studies show that the risks of term antepartum stillbirth rise gradually from 37 weeks and is highest beyond 41 weeks. Rates of neonatal mortality and severe morbidity in contrast, are highest at 37 weeks, reach a nadir at 39 weeks before rising again. Collectively, available observational data,^41^, ^42^, ^43^, ^44^, ^45^, ^46^, ^47^, ^48^ Cochrane 2020 review,^49^ evidence from the ARRIVE trial^14^ and the results of our study suggest that planned birth at 39 weeks reduces perinatal mortality and other adverse outcomes although the magnitude of risk reduction varies significantly between studies. Although these data support the ACOG/SMFM advice that it is reasonable to offer planned birth by induction of labour to low-risk, nulliparous women at 39^+0^–39^+6^ weeks it is unclear if it is also reasonable to offer these women planned birth by scheduled caesarean section.

Our findings that women who underwent induction of labour had lower rates of caesarean birth are also consistent with the ARRIVE trial^14^ and the results of a recent meta-analysis^12^ showing that induction of labour at 39 weeks was associated with a lower relative risk of caesarean section (RR 0.83; 95% CI 0.74–0.93). There are however concerns that induction of labour may increase the risk of intrapartum interventions.^50^

Unlike a randomised trial where women are allocated to induction of labour or expectant management, a policy of offering induction of labour to women at 39 weeks will no doubt result in some women requesting a scheduled caesarean section instead at the same gestation.^19^^,^^51^ Therefore, a strength of our study is our inclusion of real-world data of women undergoing induction of labour and those having scheduled caesarean section. Based on this we were able to assess the individual impact of both interventions on outcomes. Although overall, planned birth was associated with a significant reduction in the odds of antepartum and intrapartum stillbirth, neither induction of labour nor scheduled caesarean section individually had a significant impact on these outcomes. Conversely, the odds for severe neonatal neurological and non-neurological morbidity were lower for both induction of labour and scheduled caesarean section. Given ongoing placental senescence and the greater likelihood of pregnancy complications such as pre-eclampsia with increasing gestation^52^, ^53^, ^54^, ^55^ which increases the risk of adverse perinatal and maternal outcomes particularly the risk of foetal neurological injury antenatally and during labour, it is perhaps not surprising that planned birth at 39 weeks appeared to reduces the risk of severe neonatal neurological injury compared to infants born at a later gestational age. As most severe neonatal non neurological complications are related to birth trauma secondary to complicated or instrumental vaginal births, the possible beneficial effect associated with planned birth at 39^+0^–39^+6^ weeks in our study might be mediated by a reduction in instrumental vaginal births and other complications of attempted vaginal birth such as shoulder dystocia because of smaller infant size at the earlier gestation.

Strengths of this study are its size^56^ and focus on low-risk women. We were also guided by the principles of the target trial emulation framework and clearly defined “time zero” for our population was 39^+0^ weeks for both expectant management and planned birth groups.^57^^,^^58^ An important distinction between our study and other publications is our stratification of outcomes by method of planned birth (i.e., induction of labour or scheduled caesarean section) thus enabling direct comparison and evaluation of potential benefit and risk. Secondly, we performed subgroup analysis by parity and infant size because both are important factors frequently encountered in clinical practice influencing perinatal and maternal outcomes. Concerns about infant size in particular, are frequently cited as justification for the timing and mode of birth, as both small and large for gestational age infants are at increased risk of adverse perinatal outcomes.^59^^,^^60^ Thirdly, our study outcomes—for which we had complete ascertainment^56^—reflect severe consequences impacting both the woman and her infant. Our choice of components for the composite perinatal outcomes was guided by recent publications^61^^,^^62^ which defined a core perinatal outcome set identified as important and meaningful by key international stakeholders including parents, midwives, nurses and allied health professionals, obstetricians, neonatologists, paediatricians, academics, and researchers. These outcomes are applicable to any research involving infants in a high-income setting and are intended to apply regardless of gestational age at birth, birth weight, illness severity, specific infant population, clinical setting, or condition. We also assessed the risks of maternal-infant separation, an outcome rated as extremely important by women and families.^63^, ^64^, ^65^ We adjusted for clinically relevant confounders, considered potential associations with changes in practice by adjusting for the year of birth and stratified by effect measure modifiers. We however did not adjust for multiple comparisons, which could increase the risk of false positive results. Finally, our various sensitivity analyses where we included 138,614 women that spontaneously laboured at 39^+0^–39^+6^ weeks and 85,942 women that possibly developed a subsequent high-risk complication (hypertension, or diabetes mellitus or antepartum haemorrhage) (Fig. 1, Supplementary Figures S2 and S3, Supplementary Table S6) showed similar results to our primary analyses, confirming the apparent benefit associated with planned birth for perinatal mortality, severe neonatal neurological morbidity, severe neonatal non-neurological morbidity, severe perineal trauma, shoulder dystocia, and caesarean birth.

Limitations of this study include our inability to account for the effects of unmeasured confounding or selection bias, which can lead to artificially extreme p values in observational studies compared with randomised studies. We also could not completely account for the potential effects of interventions or treatment or changes in clinical practice over the study period. Treatment effect may have had a more pronounced effect at the extremes of birthweight where interventions such as planned birth, mode of birth, and intensity of foetal surveillance may have altered outcomes. Importantly, although women in the planned birth group had an indication for intervention, we were not able to completely discount the possibility of other undocumented indications which may have influenced outcomes. However, this would result in more conservative estimates of benefit—if women in the planned delivery group were delivered for additional clinical indications that we were unable to ascertain, outcomes as a group would be expected to be worse than women with expectant management without such conditions. Conversely, it is also possible that some women were delivered for non-clinical reasons. Here we aimed to strike a balance between excluding all women with any possible risk factors (which would be divorced from clinical reality) while excluding women with important risk factors (diabetes mellitus, hypertension and other medical co-morbidities, adolescents, or age >40 years or raised body mass index). This *a priori* decision we felt was reasonable given the relatively high prevalence of these conditions during pregnancy and their strong association with adverse outcomes. Our use of a historic data set, albeit one with prospectively entered data, is potentially limited by temporal variations or errors in coding practice and missing or incorrectly entered information including gestational age, all of which could potentially have influenced data quality and stability over the study period. We assumed that gestational age was correctly ascertained at the individual patient level, accurately recorded and reported. However, as this was a retrospective observational study of routinely collected information we accept the inherent limitations of this assumption. We confirmed the veracity of data by multiple means including a rigorous process of repeated cycles of screening and identifying and subsequent editing of unclear data. Finally, the issue of how to handle women in the expectant management group who spontaneously laboured at 39^+0^ and 39^+6^ weeks and delivered within the same week deserves mention. We adopted the approach many other investigators^66^, ^67^, ^68^, ^69^, ^70^ took and excluded this group of women from our primary analysis. In secondary analyses including these women the point estimates for the various outcomes changed slightly but remained consistent thus supporting the overall conclusions of our study. Overall, these various limitations corroborate our main conclusion that adequately powered randomised controlled trials are needed.

What are some clinical implications of our study? Firstly, we stress that the results of this observational study should not be taken as unqualified support for a policy of routinely offering planned birth (particularly by scheduled caesarean section) at 39^+0^–39^+6^ weeks in all low-risk women. Pending definitive evidence from randomised trials, our finding that planned birth is associated with better perinatal and maternal outcomes may provide reassurance to women and reduce requests for non-medically indicated early term birth. Secondly, although scheduled caesarean section is associated with a greater reduction in odds of some adverse outcomes when compared to induction of labour, further evidence is required before changing practice. The inherent maternal risks of caesarean birth (haemorrhage and blood transfusion, visceral injury, surgical site infection, post-surgery thromboembolic complications, hysterectomy, and maternal death) will continue to influence obstetric reticence for routinely offering this option^23^^,^^71^ particularly as maternal and infant outcomes following caesarean section differ by indication, urgency, and whether birth takes place in low- or high-resource settings.^71^ The longer-term impact on maternal and offspring health also needs assessment. Finally, health economic considerations are important. A sub-study of 1201 women enrolled in the ARRIVE trial, showed that the relative direct healthcare costs of maternal and neonatal care (up to 8 weeks postpartum) were no different for those undergoing planned induction of labour compared to expectant management (mean difference +4.7%; 95% CI −2.1% to +12.0%; p = 0.18).^72^ Other studies also show that compared to expectant management, healthcare costs are lower for induction of labour.^73^ Following publication of the ARRIVE trial, induction of labour rates in the United States increased with only a modest decrease in caesarean delivery rates.^74^ Clearly, if a policy of planned birth aims solely to reduce perinatal mortality, then very large numbers of either induction of labour or scheduled caesarean section would be required. However, if the goal is a reduction in severe neonatal morbidity or adverse maternal outcome, or a composite of perinatal mortality or any severe neonatal morbidity, then far fewer numbers would be required, and the health economic justification may be more reasonable.

In conclusion, this study provides detailed information of outcomes for low-risk women, families, and their caregivers, when considering the options of planned birth compared to continuing the pregnancy. Planned birth at 39^+0^–39^+6^ weeks was associated with lower odds of perinatal mortality, severe neonatal morbidity, maternal complications, severe perineal trauma, shoulder dystocia, and caesarean birth. Scheduled caesarean section was associated with greater reduction in odds of adverse outcomes compared to induction of labour. This information may be helpful in counselling low-risk women when deciding the appropriate mode of and gestation at birth. However, obstetric interventions, particularly routine planned birth, often results in polarised views^75^, ^76^, ^77^ amongst healthcare providers and women. Thus, robust evidence from adequately powered prospective trials in different countries, health economic settings and populations are required to assess the cost implications, safety, efficacy, and acceptability (including patient reported outcomes) of a policy of planned birth at 39 weeks in low-risk women before any change in practice is instituted.

## Contributors

SK conceived the study, provided overall supervision, reviewed and revised all versions of the manuscript. KC and SK wrote the first draft. KC accessed the data from the Queensland perinatal dataset and both SK and KC verified the data. KC analysed the data with input from all authors. WTM provided significant methodological input. All authors contributed to writing of the manuscript and had final responsibility to submit for publication.

## Data sharing statement

All code, scripts and data used to produce the results in this article may be available to other researchers provided appropriate ethics approval, inter-institutional data sharing agreements and other regulatory requirements are in place. Additional specific approval from the Data Custodian of the Statistical Services Branch of Queensland's Department of Health will also be required. Approval for some of these requirements are outside the remit of the authors.

## Declaration of interests

No conflicts of interests are reported.
