# Supplementary material for: Perinatal mortality and other severe adverse outcomes following planned birth at 39 weeks versus expectant management in low-risk women: a population based cohort study
Source: eClinicalMedicine. 2025 Jan 25;80:103076. doi: 10.1016/j.eclinm.2025.103076 (PMC11803212; doi:10.1016/j.eclinm.2025.103076)
Supplement: Supplementary Figures and Tables [file mmc1.docx]

**
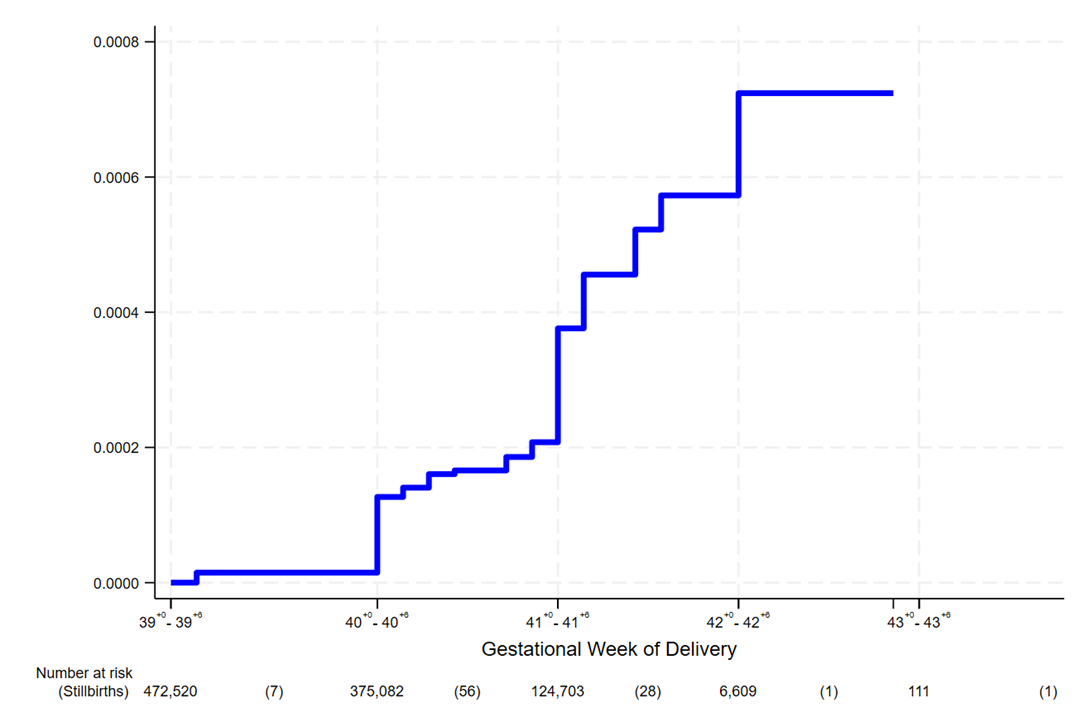
**

**Supplementary Figure 1**. Kaplan Meier curve for the probability of antepartum stillbirth by gestational age. **Population is pregnancies with a live infant at 39^+0^ weeks.*

| **Adjusted Odds Ratios (95% Confidence Intervals)** | | |
| --- | --- | --- |
| 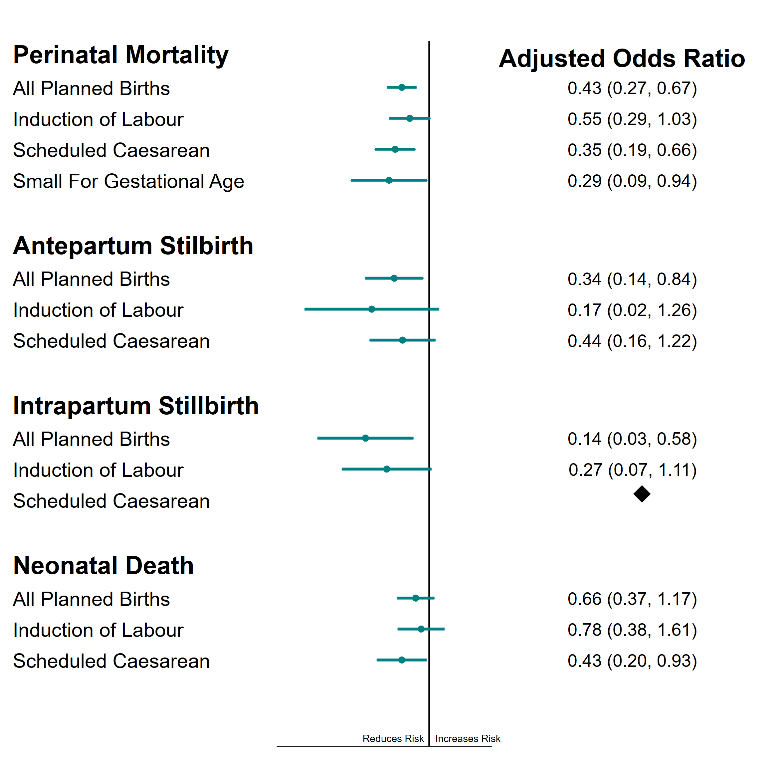 | 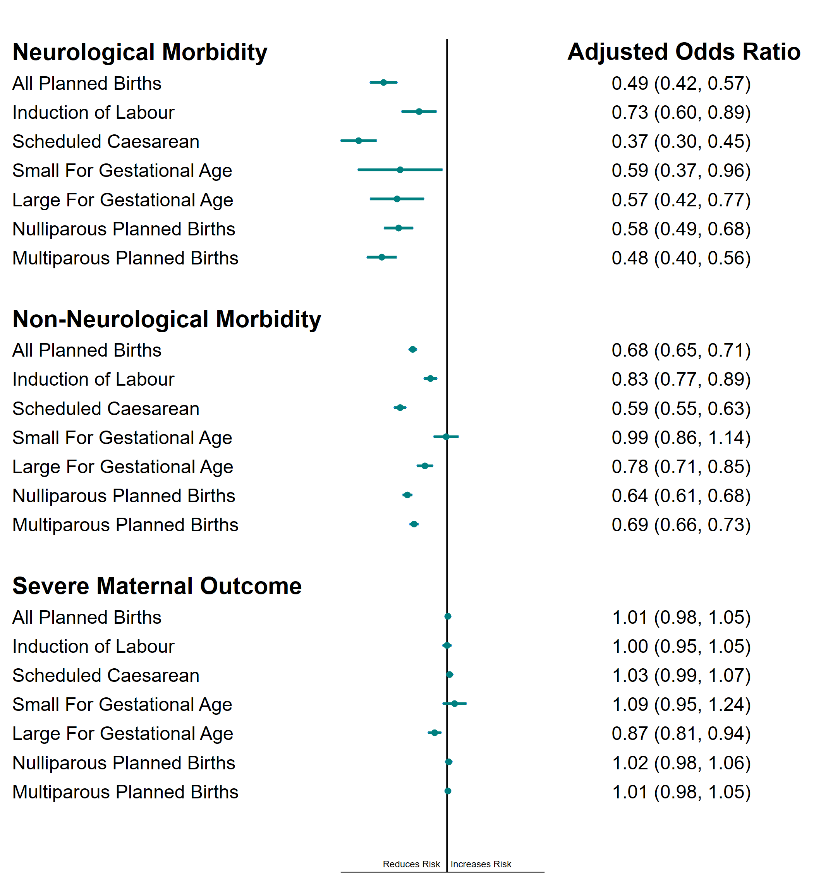 | 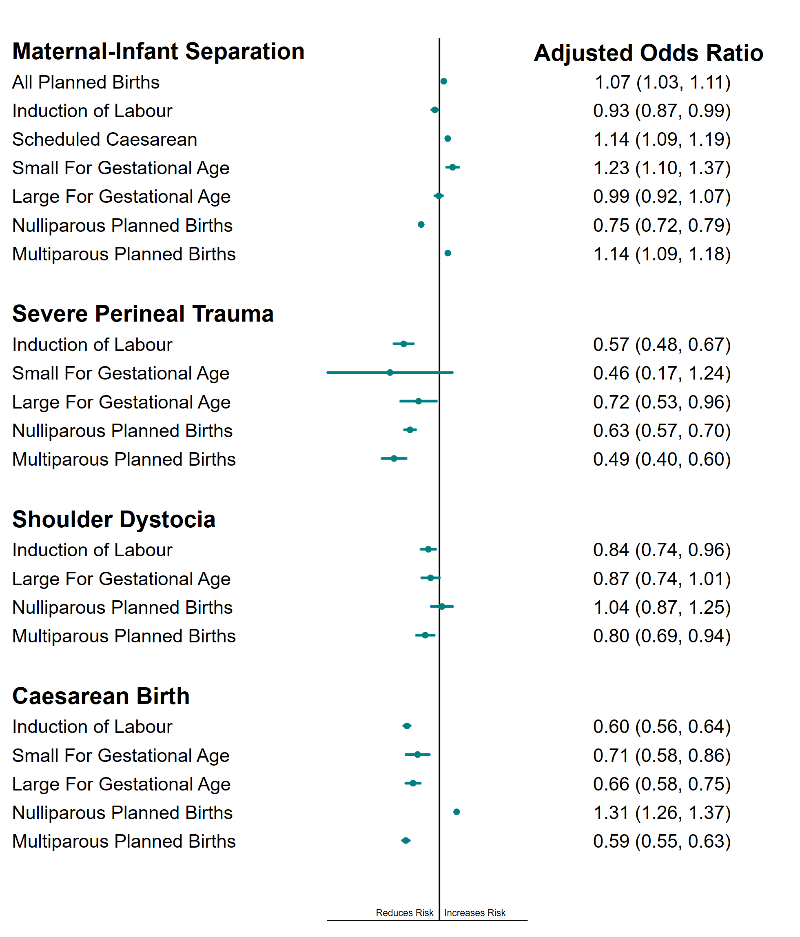 |

Perinatal Mortality: Antepartum Stillbirth, Intrapartum Stillbirth, Neonatal Death; Severe Perineal Trauma: Grades 3 or 4 Perineal Tears; ♦No Intrapartum stillbirths observed with elective caesarean section

**Supplementary Figure 2. Sensitivity analysis including pregnancies with spontaneous labour between 39^+0^ and 39^+6^ weeks: Adjusted odds ratios from multivariable logistic regression models of planned birth at 39^+0^ to 39^+6^ weeks compared to expectant management for study outcomes.**

| **Adjusted Odds Ratios (95% Confidence Intervals)** | | |
| --- | --- | --- |
| 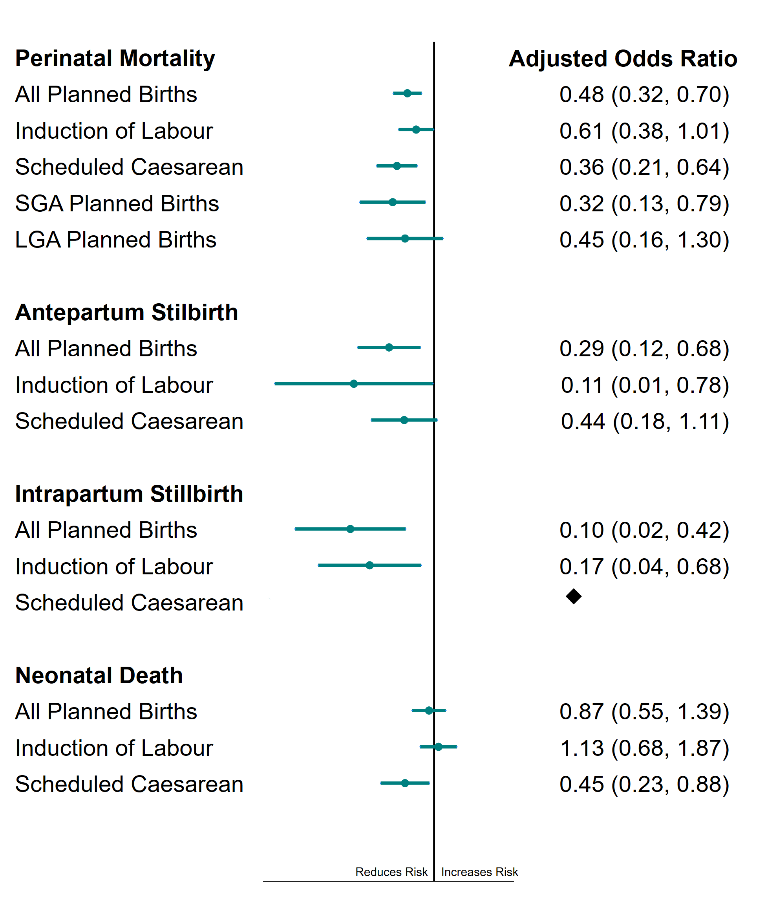 | 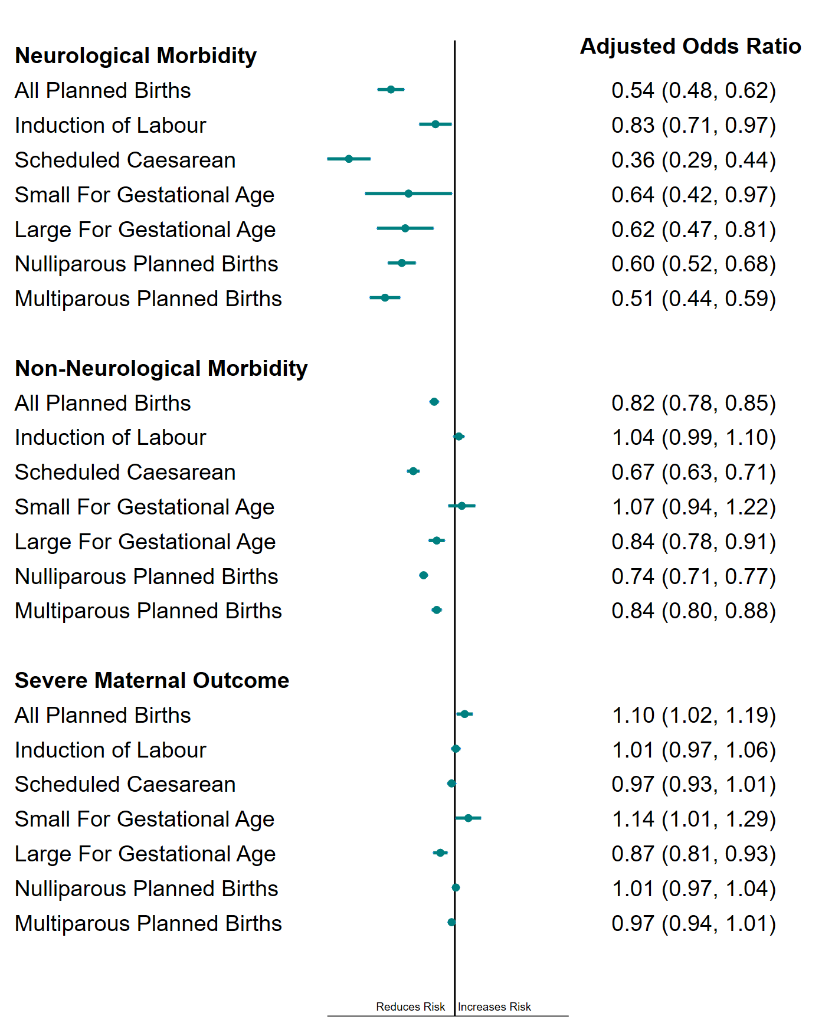 | 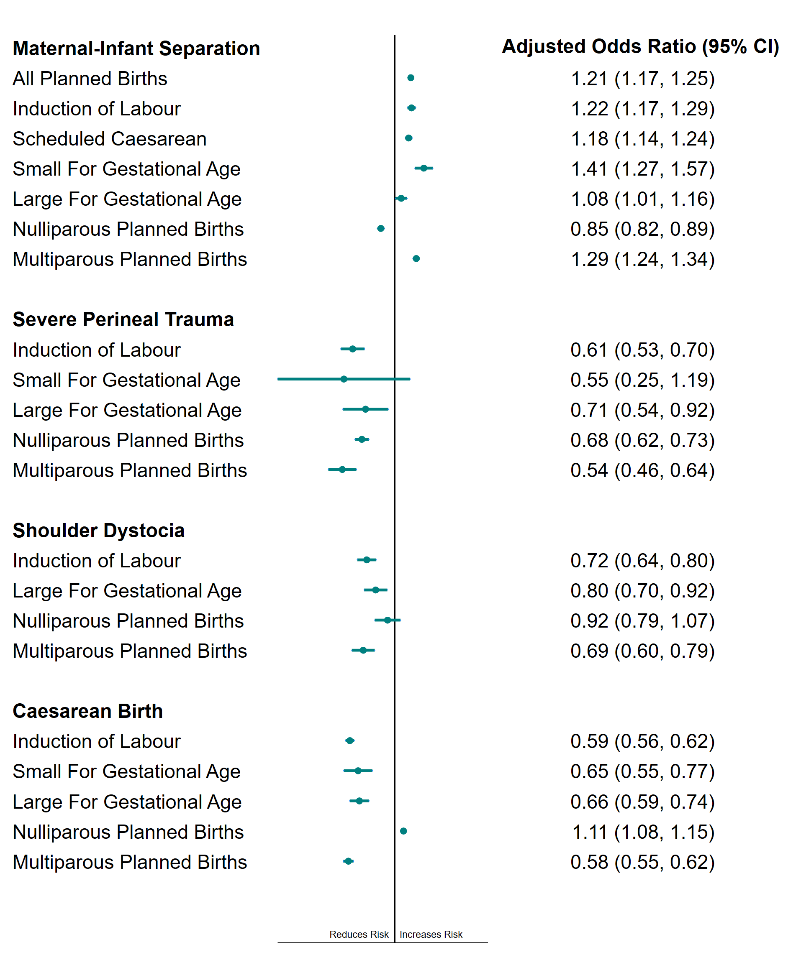 |

Perinatal Mortality: Antepartum Stillbirth, Intrapartum Stillbirth, Neonatal Death; Severe Perineal Trauma: Grades 3 or 4 Perineal Tears; ♦No Intrapartum stillbirths observed with elective caesarean section

**Supplementary Figure 3. Sensitivity analysis including pregnancies complicated by hypertension and/or diabetes mellitus and/or antepartum haemorrhage: Adjusted odds ratios from multivariable logistic regression models of planned birth at 39^+0^ to 39^+6^ weeks compared to expectant management for study outcomes.** **Note that not all pregnancies initially excluded because of antepartum haemorrhage, diabetes mellitus or hypertension were analysed in this sensitivity analyses because they may also have been excluded for having other exclusion criteria (maternal age, BMI, antepartum stillbirth etc.).*

**Supplementary Table 1: ICD-10 codes used in this study**

| **Hypertension** |  |
| --- | --- |
| I10 | Essential (primary) hypertension |
| I110 | Hypertensive heart disease with (congestive) heart failure |
| I119 | Hypertensive heart disease without (congestive) heart failure |
| I120 | Hypertensive renal disease with renal failure |
| I129 | Hypertensive renal disease without renal failure |
| I130 | Hypertensive heart and renal disease with (congestive) heart failure |
| I131 | Hypertensive heart and kidney disease with kidney failure |
| I139 | Hypertensive heart and kidney disease, unspecified |
| I150 | Renovascular hypertension |
| I151 | Hypertension secondary to other renal disorders |
| I152 | Hypertension secondary to endocrine disorders |
| I158 | Other secondary hypertension |
| I159 | Secondary hypertension, unspecified |
| I272 | Other secondary pulmonary hypertension |
| O10 | Pre-existing hypertension in pregnancy, childbirth and the puerperium |
| O100 | Pre-existing essential hypertension complicating pregnancy, childbirth and the puerperium |
| O102 | Pre-existing hypertensive renal disease complicating pregnancy, childbirth and the puerperium |
| O103 | Pre-existing hypertensive heart and kidney disease complicating pregnancy, childbirth and the puerperium |
| O104 | Pre-existing secondary hypertension complicating pregnancy, childbirth and the puerperium |
| O109 | Unspecified pre-existing hypertension complicating pregnancy, childbirth and the puerperium |
| R030 | Elevated blood-pressure reading, without diagnosis of hypertension |
|  |  |
| O13 | Gestational [pregnancy-induced] hypertension without significant proteinuria |
| O16 | Unspecified maternal hypertension |
| O120 | Gestational oedema |
| P000 | Fetus and newborn affected by maternal hypertensive disorders |
| O121 | Gestational proteinuria |
| O122 | Gestational oedema with proteinuria |
| O140 | Mild to moderate pre-eclampsia |
| O140 | Moderate pre-eclampsia |
| O141 | Severe pre-eclampsia |
| O142 | HELLP syndrome |
| O149 | Pre-eclampsia, unspecified |
| O150 | Eclampsia in pregnancy |
| O151 | Eclampsia in labour |
| O159 | Eclampsia, unspecified as to time period |
|  |  |
| **Diabetes** |  |
| E1011 | Type 1 diabetes mellitus with ketoacidosis, without coma |
| E1015 | Type 1 diabetes mellitus with ketoacidosis, with lactic acidosis, without coma |
| E1021 | Type 1 diabetes mellitus with incipient diabetic nephropathy |
| E1022 | Type 1 diabetes mellitus with established diabetic nephropathy |
| E1023 | Type 1 diabetes mellitus with end-stage renal disease [ESRD] |
| E1029 | Type 1 diabetes mellitus with other specified kidney complication |
| E1031 | Type 1 diabetes mellitus with background retinopathy |
| E1033 | Type 1 diabetes mellitus with proliferative retinopathy |
| E1034 | Type 1 diabetes mellitus with other retinopathy |
| E1036 | Type 1 diabetes mellitus with diabetic cataract |
| E1040 | Type 1 diabetes mellitus with unspecified neuropathy |
| E1041 | Type 1 diabetes mellitus with diabetic mononeuropathy |
| E1042 | Type 1 diabetes mellitus with diabetic polyneuropathy |
| E1043 | Type 1 diabetes mellitus with diabetic autonomic neuropathy |
| E1049 | Type 1 diabetes mellitus with other specified neurological complication |
| E1051 | Type 1 diabetes mellitus with peripheral angiopathy, without gangrene |
| E1061 | Type 1 diabetes mellitus with specified diabetic musculoskeletal and connective tissue complication |
| E1064 | Type 1 diabetes mellitus with hypoglycaemia |
| E1065 | Type 1 diabetes mellitus with poor control |
| E1069 | Type 1 diabetes mellitus with other specified complication |
| E1071 | Type 1 diabetes mellitus with multiple microvascular complications |
| E1073 | Type 1 diabetes mellitus with foot ulcer due to multiple causes |
| E108 | Type 1 diabetes mellitus with unspecified complicatio |
| E109 | Type 1 diabetes mellitus without complication |
| O240 | Pre-existing diabetes mellitus, Type 1, in pregnancy |
|  |  |
| E1101 | Type 2 diabetes mellitus with hyperosmolarity without nonketotic hyperglycaemic-hyperosmolar coma [NKHHC] |
| E1111 | Type 2 diabetes mellitus with ketoacidosis, without coma |
| E1121 | Type 2 diabetes mellitus with incipient diabetic nephropathy |
| E1122 | Type 2 diabetes mellitus with established diabetic nephropathy |
| E1131 | Type 2 diabetes mellitus with background retinopathy |
| E1134 | Type 2 diabetes mellitus with other retinopathy |
| E1136 | Type 2 diabetes mellitus with diabetic cataract |
| E1140 | Type 2 diabetes mellitus with unspecified neuropathy |
| E1142 | Type 2 diabetes mellitus with diabetic polyneuropathy |
| E1143 | Type 2 diabetes mellitus with diabetic autonomic neuropathy |
| E1153 | Type 2 diabetes mellitus with diabetic ischaemic cardiomyopathy |
| E1161 | Type 2 diabetes mellitus with specified diabetic musculoskeletal and connective tissue complication |
| E1162 | Type 2 diabetes mellitus with specified skin and subcutaneous tissue complication |
| E1164 | Type 2 diabetes mellitus with hypoglycaemia |
| E1165 | Type 2 diabetes mellitus with poor control |
| E1171 | Type 2 diabetes mellitus with multiple microvascular and other specified nonvascular complications |
| E1172 | Type 2 diabetes mellitus with features of insulin resistance |
| E1173 | Type 2 diabetes mellitus with foot ulcer due to multiple causes |
| E118 | Type 2 diabetes mellitus with unspecified complication |
| E119 | Type 2 diabetes mellitus without complication |
| O241 | Pre-existing diabetes mellitus, Type 2, in pregnancy |
| O2411 | Pre-existing diabetes mellitus, Type 2, in pregnancy, non-insulin treated |
| O2412 | Pre-existing diabetes mellitus, Type 2, in pregnancy, insulin treated |
| O2413 | Pre-existing diabetes mellitus, Type 2, in pregnancy, oral hypoglycaemic therapy |
| O2414 | Pre-existing diabetes mellitus, Type 2, in pregnancy, other |
| O2419 | Pre-existing diabetes mellitus, Type 2, in pregnancy, unspecified |
|  |  |
| E1322 | Other specified diabetes mellitus with established diabetic nephropathy |
| E1329 | Other specified diabetes mellitus with other specified renal complication |
| E1331 | Other specified diabetes mellitus with background retinopathy |
| E1342 | Other specified diabetes mellitus with diabetic polyneuropathy |
| E1364 | Other specified diabetes mellitus with hypoglycaemia |
| E1365 | Other specified diabetes mellitus with poor control |
| E1371 | Other specified diabetes mellitus with multiple microvascular complications |
| E1372 | Other specified diabetes mellitus with features of insulin resistance |
| E138 | Other specified diabetes mellitus with unspecified complication |
| E139 | Other specified diabetes mellitus without complication |
| E1439 | Unspecified diabetes mellitus with other specified ophthalmic complication |
| E1443 | Unspecified diabetes mellitus with diabetic autonomic neuropathy |
| E1464 | Unspecified diabetes mellitus with hypoglycaemia |
| E149 | Unspecified diabetes mellitus without complication |
| G590 | Diabetic mononeuropathy |
| O242 | Pre-existing diabetes mellitus, other specified type, in pregnancy |
| O2422 | Pre-existing diabetes mellitus, other specified type, in pregnancy, insulin treated |
| O2423 | Pre-existing diabetes mellitus, other specified type, in pregnancy, oral hypoglycaemic therapy |
| O2424 | Pre-existing diabetes mellitus, other specified type, in pregnancy, other |
| O2429 | Pre-existing diabetes mellitus, other specified type, in pregnancy, unspecified |
| O243 | Pre-existing diabetes mellitus, unspecified, in pregnancy |
| O2431 | Pre-existing diabetes mellitus, unspecified, in pregnancy, non-insulin treated |
| O2432 | Pre-existing diabetes mellitus, unspecified, in pregnancy, insulin treated |
| O2433 | Pre-existing diabetes mellitus, unspecified type, in pregnancy, oral hypoglycaemic therapy |
| O2434 | Pre-existing diabetes mellitus, unspecified type, in pregnancy, other |
| O2439 | Pre-existing diabetes mellitus, unspecified, in pregnancy, unspecified |
| O2453 | Pre-existing intermediate hyperglycaemia, in pregnancy, oral hypoglycaemic therapy |
| O2459 | Pre-existing impaired glucose regulation, in pregnancy, unspecified |
| O2508 | Diabetes mellitus — Diabetes with other specified manifestations, type |
| O2509 | "Diabetes mellitus — With unspecified complication" |
| P701 | Syndrome of infant of a diabetic mother |
| O2441 | Diabetes mellitus arising in pregnancy, non-insulin-requiring |
| O2442 | Diabetes mellitus arising in pregnancy, insulin-requiring |
| O2443 | Diabetes mellitus arising during pregnancy, oral hypoglycaemic therapy |
| O2444 | Diabetes mellitus arising during pregnancy, other |
| O2449 | Diabetes mellitus arising at or after 24 weeks gestation, unspecified |
| O249 | Diabetes mellitus in pregnancy, unspecified |
| O2492 | Diabetes mellitus in pregnancy, unspecified onset, insulin treated |
| O2493 | Diabetes mellitus in pregnancy, unspecified onset, oral hypoglycaemic therapy |
| O2499 | Diabetes mellitus in pregnancy, unspecified onset, unspecified |
| O244 | Diabetes mellitus arising in pregnancy |
| P700 | Syndrome of infant of mother with gestational diabetes |
|  |  |
| **Antepartum haemorrhage** |  |
| O441 | Placenta praevia with haemorrhage |
| O432 | Morbidly adherent placenta |
| O450 | Premature separation of placenta with coagulation defect |
| O458 | Other premature separation of placenta |
| O459 | Premature separation of placenta, unspecified |
| O460 | Antepartum haemorrhage with coagulation defect |
| O468 | Other antepartum haemorrhage |
| O469 | Antepartum haemorrhage, unspecified |
| P021 | Fetus and newborn affected by other forms of placental separation and haemorrhage |
|  |  |
| **Postpartum Haemmorrhage** |  |
|  |  |
| D62 | Acute posthaemorrhagic anaemia |
| D683 | Haemorrhagic disorder due to circulating anticoagulants |
| D698 | Other specified haemorrhagic conditions |
| D698 | Other specified haemorrhagic conditions |
| D699 | Haemorrhagic condition, unspecified |
| O720 | Third-stage haemorrhage |
| O721 | Other immediate postpartum haemorrhage |
| O722 | Delayed and secondary postpartum haemorrhage |
| O723 | Postpartum coagulation defects |
|  |  |
| **Postpartum sepsis** |  |
| O85 | Puerperal sepsis |
| O860 | Infection of obstetric surgical wound |
| O861 | Other infection of genital tract following delivery |
| O862 | Urinary tract infection following delivery |
| O863 | Other genitourinary tract infections following delivery |
| O864 | Pyrexia of unknown origin following delivery |
| O868 | Other specified puerperal infections |
| O9110 | Abscess of breast associated with childbirth, without mention of attachment difficulty |
| O9111 | Abscess of breast associated with childbirth, with mention of attachment difficulty |
|  |  |
| **Neurological morbidity** |  |
| P110 | Cerebral oedema due to birth injury |
| P111 | Other specified brain damage due to birth injury |
| P112 | Unspecified brain damage due to birth injury |
| P200 | Intrauterine hypoxia first noted before onset of labour |
| P201 | Intrauterine hypoxia first noted during labour and delivery |
| P209 | Intrauterine hypoxia, unspecified |
| P210 | Severe birth asphyxia |
| P211 | Mild and moderate birth asphyxia |
| P219 | Birth asphyxia, unspecified |
| P90 | Convulsions of newborn |
| P910 | Neonatal cerebral ischaemia |
| P916 | Hypoxic ischaemic encephalopathy [HIE] of newborn |
| P9160 | Hypoxic ischaemic encephalopathy [HIE] of newborn, unspecified |
| P9161 | Stage 1 hypoxic ischaemic encephalopathy [HIE] of newborn |
| P9162 | Stage 2 hypoxic ischaemic encephalopathy [HIE] of newborn |
| P9163 | Stage 3 hypoxic ischaemic encephalopathy [HIE] of newborn |
| P9181 | Neonatal encephalopathy |
|  |  |
| P916 | Hypoxic ischaemic encephalopathy [HIE] of newborn |
| P9160 | Hypoxic ischaemic encephalopathy [HIE] of newborn, unspecified |
| P9163 | Stage 3 hypoxic ischaemic encephalopathy [HIE] of newborn |
| P9162 | Stage 2 hypoxic ischaemic encephalopathy [HIE] of newborn |
| P9161 | Stage 1 hypoxic ischaemic encephalopathy [HIE] of newborn |
| P910 | Neonatal cerebral ischaemia |
| P9181 | Neonatal encephalopathy |
| P90 | Neonatal convulsions |
| P520 | Intraventricular (nontraumatic) haemorrhage, grade 1, of fetus and newborn |
| P521 | Intraventricular (nontraumatic) haemorrhage, grade 2, of fetus and newborn |
| P522 | Intraventricular (nontraumatic) haemorrhage, grade 3, of fetus and newborn |
| P523 | Unspecified intraventricular (nontraumatic) haemorrhage of fetus and newborn |
| P524 | Intracerebral (nontraumatic) haemorrhage of fetus and newborn |
| P525 | Subarachnoid (nontraumatic) haemorrhage of fetus and newborn |
| P528 | Other intracranial (nontraumatic) haemorrhages of fetus and newborn |
| P529 | Intracranial (nontraumatic) haemorrhage of fetus and newborn, unspecified |
|  |  |
| **Sepsis** |  |
| A020 | Salmonella enteritis |
| A045 | Campylobacter enteritis |
| A048 | Other specified bacterial intestinal infections |
| A080 | Rotaviral enteritis |
| A082 | Adenoviral enteritis |
| A084 | Viral intestinal infection, unspecified |
| A09 | Diarrhoea and gastroenteritis of presumed infectious origin |
| A099 | Gastroenteritis and colitis of unspecified origin |
| A288 | Other specified zoonotic bacterial diseases, not elsewhere classified |
| A370 | Whooping cough due to Bordetella pertussis |
| A379 | Whooping cough, unspecified |
| A390 | Meningococcal meningitis |
| A401 | Septicaemia due to streptococcus, group B |
| A402 | Sepsis due to streptococcus, group D and enterococcus |
| A403 | Septicaemia due to Streptococcus pneumoniae |
| A411 | Sepsis due to other specified staphylococcus |
| A412 | Septicaemia due to unspecified staphylococcus |
| A413 | Septicaemia due to Haemophilus influenzae |
| A4152 | Sepsis due to Pseudomonas |
| A4158 | Sepsis due to other Gram-negative organisms |
| A419 | Septicaemia, unspecified |
| A490 | Staphylococcal infection, unspecified |
| A4900 | Staphylococcal infection, unspecified site |
| A4901 | Staphylococcus aureus infection, unspecified site |
| A491 | Streptococcal infection, unspecified |
| A492 | Haemophilus influenzae infection, unspecified site |
| A493 | Mycoplasma infection, unspecified site |
| A498 | Other bacterial infections of unspecified site |
| A499 | Bacterial infection, unspecified |
| A500 | Early congenital syphilis, symptomatic |
| A502 | Early congenital syphilis, unspecified |
| A509 | Congenital syphilis, unspecified |
| A851 | Adenoviral encephalitis |
| A86 | Unspecified viral encephalitis |
| A870 | Enteroviral meningitis (G02.0*) |
| A878 | Other viral meningitis |
| A879 | Viral meningitis, unspecified |
| B004 | Herpesviral encephalitis |
| B005 | Herpesviral ocular disease |
| B007 | Disseminated herpesviral disease |
| B009 | Herpesviral infection, unspecified |
| B019 | Varicella without complication |
| B259 | Cytomegaloviral disease, unspecified |
| B332 | Viral carditis |
| B341 | Enterovirus infection, unspecified site |
| B343 | Parvovirus infection, unspecified |
| B349 | Viral infection, unspecified |
| B369 | Superficial mycosis, unspecified |
| B370 | Candidal stomatitis |
| B372 | Candidiasis of skin and nail |
| B377 | Candidal septicaemia |
| B379 | Candidiasis, unspecified |
| B389 | Coccidioidomycosis, unspecified |
| B390 | Acute pulmonary histoplasmosis capsulati |
| B432 | Subcutaneous phaeomycotic abscess and cyst |
| B49 | Unspecified mycosis |
| B951 | Streptococcus, group B, as the cause of diseases classified to other chapters |
| B952 | Streptococcus, group D, as the cause of diseases classified to other chapters |
| B954 | Other streptococcus as the cause of diseases classified to other chapters |
| B9542 | Streptococcus, Group G |
| B9548 | Streptococcus, other specified group |
| B955 | Unspecified streptococcus as the cause of diseases classified to other chapters |
| B956 | Staphylococcus aureus as the cause of diseases classified to other chapters |
| B957 | Other staphylococcus as the cause of diseases classified to other chapters |
| B958 | Unspecified staphylococcus as the cause of diseases classified to other chapters |
| B961 | Klebsiella pneumoniae [K. pneumoniae] as the cause of diseases classified to other chapters |
| B962 | Escherichia coli [E. coli] as the cause of diseases classified to other chapters |
| B9639 | Haemophilus influenzae [H. influenzae] type not specified, as the cause of diseases classified to other chapters |
| B965 | Pseudomonas (aeruginosa) as the cause of diseases classified to other chapters |
| B966 | Bacillus fragilis [B. fragilis] as the cause of diseases classified to other chapters |
| B967 | Clostridium perfringens [C. perfringens] as the cause of diseases classified to other chapters |
| B968 | Other specified bacterial agents as the cause of diseases classified to other chapters |
| B970 | Adenovirus as the cause of diseases classified to other chapters |
| B971 | Enterovirus as the cause of diseases classified to other chapters |
| B973 | Retrovirus as the cause of diseases classified to other chapters |
| B974 | Respiratory syncytial virus as the cause of diseases classified to other chapters |
| B976 | Parvovirus as the cause of diseases classified to other chapters |
| G001 | Pneumococcal meningitis |
| G002 | Streptococcal meningitis |
| G008 | Other bacterial meningitis |
| G009 | Bacterial meningitis, unspecified |
| G039 | Meningitis, unspecified |
| G049 | Encephalitis, myelitis and encephalomyelitis, unspecified |
| G051 | Encephalitis, myelitis and encephalomyelitis in viral diseases classified elsewhere |
| G060 | Intracranial abscess and granuloma |
| G08 | Intracranial and intraspinal phlebitis and thrombophlebitis |
| I38 | Endocarditis, valve unspecified |
| I400 | Infective myocarditis |
| I514 | Myocarditis, unspecified |
| J111 | Influenza with other respiratory manifestations, virus not identified |
| J121 | Respiratory syncytial virus pneumonia |
| J13 | Pneumonia due to Streptococcus pneumoniae |
| J151 | Pneumonia due to Pseudomonas |
| J180 | Bronchopneumonia, unspecified |
| J189 | Pneumonia, unspecified |
| J210 | Acute bronchiolitis due to respiratory syncytial virus |
| J219 | Acute bronchiolitis, unspecified |
| J22 | Unspecified acute lower respiratory infection |
| J869 | Pyothorax without fistula |
| K612 | Anorectal abscess |
| K631 | Perforation of intestine (nontraumatic) |
| K750 | Abscess of liver |
| L028 | Cutaneous abscess, furuncle and carbuncle of other sites |
| L0302 | Cellulitis of toe |
| L0311 | Cellulitis of lower limb |
| L033 | Cellulitis of trunk |
| L038 | Cellulitis of other sites |
| L039 | Cellulitis, unspecified |
| L089 | Local infection of skin and subcutaneous tissue, unspecified |
| M0096 | Pyogenic arthritis, unspecified, lower leg |
| P230 | Congenital pneumonia due to viral agent |
| P231 | Congenital pneumonia due to Chlamydia |
| P232 | Congenital pneumonia due to staphylococcus |
| P233 | Congenital pneumonia due to streptococcus, group B |
| P234 | Congenital pneumonia due to Escherichia coli |
| P235 | Congenital pneumonia due to Pseudomonas |
| P236 | Congenital pneumonia due to other bacterial agents |
| P238 | Congenital pneumonia due to other organisms |
| P239 | Congenital pneumonia, unspecified |
| P240 | Neonatal aspiration of meconium |
| P249 | Neonatal aspiration syndrome, unspecified |
| P350 | Congenital rubella syndrome |
| P351 | Congenital cytomegalovirus infection |
| P352 | Congenital herpesviral [herpes simplex] infection |
| P353 | Congenital viral hepatitis |
| P358 | Other congenital viral diseases |
| P359 | Congenital viral disease, unspecified |
| P360 | Sepsis of newborn due to streptococcus, group B |
| P361 | Sepsis of newborn due to other and unspecified streptococci |
| P362 | Sepsis of newborn due to Staphylococcus aureus |
| P363 | Sepsis of newborn due to other and unspecified staphylococci |
| P364 | Sepsis of newborn due to Escherichia coli |
| P365 | Sepsis of newborn due to anaerobes |
| P368 | Other bacterial sepsis of newborn |
| P369 | Bacterial sepsis of newborn, unspecified |
| P371 | Congenital toxoplasmosis |
| P372 | Neonatal (disseminated) listeriosis |
| P373 | Congenital falciparum malaria |
| P375 | Neonatal candidiasis |
| P3750 | Neonatal candidiasis, unspecified |
| P3751 | Topical or gastrointestinal neonatal candidiasis |
| P3752 | Invasive neonatal candidiasis |
| P3759 | Other neonatal candidiasis |
| P378 | Other specified congenital infectious and parasitic diseases |
| P379 | Congenital infectious and parasitic disease, unspecified |
| P38 | Omphalitis of newborn with or without mild haemorrhage |
| P390 | Neonatal infective mastitis |
| P391 | Neonatal conjunctivitis and dacryocystitis |
| P392 | Intra-amniotic infection of fetus, not elsewhere classified |
| P393 | Neonatal urinary tract infection |
| P394 | Neonatal skin infection |
| P398 | Other specified infections specific to the perinatal period |
| P399 | Infection specific to the perinatal period, unspecified |
| P781 | Other neonatal peritonitis |
| R572 | Septic shock |
| T814 | Infection following a procedure, not elsewhere classified |
| Z0371 | Observation for suspected newborn infectious condition |
|  |  |
| **Necrotising Enterocolitis** |  |
| P77 | Necrotizing enterocolitis of fetus and newborn |
| P780 | Perinatal intestinal perforation |
|  |  |
| **Birth Trauma** |  |
| P100 | Subdural haemorrhage due to birth injury |
| P101 | Cerebral haemorrhage due to birth injury |
| P104 | Tentorial tear due to birth injury |
| P108 | Other intracranial lacerations and haemorrhages due to birth injury |
| P111 | Other specified brain damage due to birth injury |
| P112 | Unspecified brain damage due to birth injury |
| P113 | Birth injury to facial nerve |
| P120 | Cephalhaematoma due to birth injury |
| P121 | Chignon due to birth injury |
| P122 | Epicranial subaponeurotic haemorrhage due to birth injury |
| P123 | Bruising of scalp due to birth injury |
| P124 | Monitoring injury of scalp of newborn |
| P128 | Other birth injuries to scalp |
| P129 | Birth injury to scalp, unspecified |
| P131 | Other birth injuries to skull |
| P133 | Birth injury to other long bones |
| P134 | Fracture of clavicle due to birth injury |
| P140 | Erb's paralysis due to birth injury |
| P143 | Other brachial plexus birth injuries |
| P152 | Sternomastoid injury due to birth injury |
| P153 | Birth injury to eye |
| P154 | Birth injury to face |
| P158 | Other specified birth injuries |
| P159 | Birth injury, unspecified |
| P110 | Cerebral oedema due to birth injury |
| P115 | Birth injury to spine and spinal cord |
| P130 | Fracture of skull due to birth injury |
| P132 | Birth injury to femur |
| P138 | Birth injuries to other parts of skeleton |
| P139 | Birth injury to skeleton, unspecified |
| P141 | Klumpke's paralysis due to birth injury |
| P150 | Birth injury to liver |
| P155 | Birth injury to external genitalia |
| P103 | Subarachnoid haemorrhage due to birth injury |
| P119 | Birth injury to central nervous system, unspecified |
| P102 | Intraventricular haemorrhage due to birth trauma |
| P109 | Unspecified intracranial laceration and haemorrhage due to birth trauma |
| P156 | Subcutaneous fat necrosis due to birth trauma |
| P148 | Birth trauma to other parts of peripheral nervous system |
| S020 | Fracture of vault of skull |
| S021 | Fracture of base of skull |
| S022 | Fracture of nasal bones |
| P142 | Phrenic nerve paralysis due to birth trauma |
| P151 | Birth trauma to spleen |
| S059 | Injury of eye and orbit, unspecified |
| S065 | Traumatic subdural haemorrhage |
|  |  |
| **Hypoglycaemia** |  |
| P703 | Iatrogenic neonatal hypoglycaemia |
| P704 | Other neonatal hypoglycaemia |
|  |  |
| **Smoking, Drugs and Alcohol** |  |
| F170 | Mental and behavioural disorders due to use of tobacco, acute intoxication |
| F171 | Mental and behavioural disorders due to use of tobacco, harmful use |
| F172 | Mental and behavioural disorders due to use of tobacco, dependence syndrome |
| F173 | Mental and behavioural disorders due to use of tobacco, withdrawal state |
| F179 | Mental and behavioural disorders due to use of tobacco, unspecified mental and behavioural disorder |
| Z720 | Tobacco use, current |
| Z8643 | Personal history of tobacco use disorder |
| P042 | Fetus and newborn affected by Tobacco |
| F100 | Mental and behavioural disorders due to use of alcohol, acute intoxication |
| F101 | Mental and behavioural disorders due to use of alcohol, harmful use |
| F102 | Mental and behavioural disorders due to use of alcohol, dependence syndrome |
| F103 | Mental and behavioural disorders due to use of alcohol, withdrawal state |
| F104 | Mental and behavioural disorders due to use of alcohol, withdrawal state with delirium |
| F105 | Mental and behavioural disorders due to use of alcohol, psychotic disorder |
| F106 | Mental and behavioural disorders due to use of alcohol, amnesic syndrome |
| F108 | Mental and behavioural disorders due to use of alcohol, other mental and behavioural disorders |
| F109 | Mental and behavioural disorders due to use of alcohol, unspecified mental and behavioural disorder |
| G721 | Alcoholic myopathy |
| I426 | Alcoholic cardiomyopathy |
| O354 | Maternal care for (suspected) damage to fetus from alcohol |
| Y909 | Presence of alcohol in blood, level not specified |
| Z714 | Counselling and surveillance for alcohol use disorder |
| Z721 | Alcohol use |
| Z8641 | Personal history of alcohol use disorder |
| K292 | Alcoholic gastritis |
| K2920 | Alcoholic gastritis, without mention of haemorrhage |
| K709 | Alcoholic liver disease, unspecified |
| P043 | Fetus and newborn affected by maternal use of alcohol |
| F110 | Mental and behavioural disorders due to use of opioids, acute intoxication |
| F111 | Mental and behavioural disorders due to use of opioids, harmful use |
| F112 | Mental and behavioural disorders due to use of opioids, dependence syndrome |
| F113 | Mental and behavioural disorders due to use of opioids, withdrawal state |
| F114 | Mental and behavioural disorders due to use of opioids, withdrawal state with delirium |
| F115 | Mental and behavioural disorders due to use of opioids, psychotic disorder |
| F118 | Mental and behavioural disorders due to use of opioids, other mental and behavioural disorders |
| F119 | Mental and behavioural disorders due to use of opioids, unspecified mental and behavioural disorder |
| F120 | Mental and behavioural disorders due to use of cannabinoids, acute intoxication |
| F121 | Mental and behavioural disorders due to use of cannabinoids, harmful use |
| F122 | Mental and behavioural disorders due to use of cannabinoids, dependence syndrome |
| F123 | Mental and behavioural disorders due to use of cannabinoids, withdrawal state |
| F125 | Mental and behavioural disorders due to use of cannabinoids, psychotic disorder |
| F128 | Mental and behavioural disorders due to use of cannabinoids, other mental and behavioural disorders |
| F129 | Mental and behavioural disorders due to use of cannabinoids, unspecified mental and behavioural disorder |
| F1300 | Mental and behavioural disorders due to use of sedatives or hypnotics, acute intoxication, unspecified sedative or hypnotic |
| F1301 | Mental and behavioural disorders due to use of sedatives or hypnotics, acute intoxication, gamma hydroxybutyrate |
| F1309 | Mental and behavioural disorders due to use of sedatives or hypnotics, acute intoxication, other specified sedative or hypnotic |
| F131 | Mental and behavioural disorders due to use of sedatives or hypnotics, harmful use |
| F1310 | Mental and behavioural disorders due to use of sedatives or hypnotics, harmful use, unspecified sedative or hypnotic |
| F1311 | Mental and behavioural disorders due to use of sedatives or hypnotics, harmful use, gamma hydroxybutyrate |
| F1319 | Mental and behavioural disorders due to use of sedatives or hypnotics, harmful use, other specified sedative or hypnotic |
| F132 | Mental and behavioural disorders due to use of sedatives or hypnotics, dependence syndrome |
| F1320 | Mental and behavioural disorders due to use of sedatives or hypnotics, dependence syndrome, unspecified sedative or hypnotic |
| F133 | Mental and behavioural disorders due to use of sedatives or hypnotics, withdrawal state |
| F135 | Mental and behavioural disorders due to use of sedatives or hypnotics, psychotic disorder |
| F138 | Mental and behavioural disorders due to use of sedatives or hypnotics, other mental and behavioural disorders |
| F139 | Mental and behavioural disorders due to use of sedatives or hypnotics, unspecified mental and behavioural disorder |
| F1391 | Mental and behavioural disorders due to use of sedatives or hypnotics, unspecified mental and behavioural disorder, gamma hydroxybutyrate |
| F1399 | Mental and behavioural disorders due to use of sedatives or hypnotics, unspecified mental and behavioural disorder, other specified sedative or hypnotic |
| F141 | Mental and behavioural disorders due to use of cocaine, harmful use |
| F142 | Mental and behavioural disorders due to use of cocaine, dependence syndrome |
| F149 | Mental and behavioural disorders due to use of cocaine, unspecified mental and behavioural disorder |
| F150 | Mental and behavioural disorders due to use of other stimulants including caffeine, acute intoxication |
| F1500 | Mental and behavioural disorders due to use of other stimulants, including caffeine, acute intoxication, unspecified stimulants |
| F1501 | Mental and behavioural disorders due to use of other stimulants, including caffeine, acute intoxication, methylamphetamine |
| F1502 | Mental and behavioural disorders due to use of other stimulants, including caffeine, acute intoxication, methylenedioxy methamphetamine |
| F1509 | Mental and behavioural disorders due to use of other stimulants, including caffeine, acute intoxication, other specified stimulants |
| F151 | Mental and behavioural disorders due to use of other stimulants including caffeine, harmful use |
| F1510 | Mental and behavioural disorders due to use of other stimulants, including caffeine, harmful use, unspecified stimulants |
| F1511 | Mental and behavioural disorders due to use of other stimulants, including caffeine, harmful use, methylamphetamine |
| F1512 | Mental and behavioural disorders due to use of other stimulants, including caffeine, harmful use, methylenedioxy methamphetamine |
| F1519 | Mental and behavioural disorders due to use of other stimulants, including caffeine, harmful use, other specified stimulants |
| F152 | Mental and behavioural disorders due to use of other stimulants including caffeine, dependence syndrome |
| F1521 | Mental and behavioural disorders due to use of other stimulants, including caffeine, dependence syndrome, methylamphetamine |
| F1522 | Mental and behavioural disorders due to use of other stimulants, including caffeine, dependence syndrome, methylenedioxy methamphetamine |
| F1529 | Mental and behavioural disorders due to use of other stimulants, including caffeine, dependence syndrome, other specified stimulants |
| F153 | Mental and behavioural disorders due to use of other stimulants including caffeine, withdrawal state |
| F1530 | Mental and behavioural disorders due to use of other stimulants, including caffeine, withdrawal state, unspecified stimulants |
| F1531 | Mental and behavioural disorders due to use of other stimulants, including caffeine, withdrawal state, methylamphetamine |
| F1539 | Mental and behavioural disorders due to use of other stimulants, including caffeine, withdrawal state, other specified stimulants |
| F154 | Mental and behavioural disorders due to use of other stimulants including caffeine, withdrawal state with delirium |
| F155 | Mental and behavioural disorders due to use of other stimulants including caffeine, psychotic disorder |
| F1550 | Mental and behavioural disorders due to use of other stimulants, including caffeine, psychotic disorder, unspecified stimulants |
| F1559 | Mental and behavioural disorders due to use of other stimulants, including caffeine, psychotic disorder, other specified stimulants |
| F158 | Mental and behavioural disorders due to use of other stimulants including caffeine, other mental and behavioural disorders |
| F159 | Mental and behavioural disorders due to use of other stimulants including caffeine, unspecified mental and behavioural disorder |
| F1591 | Mental and behavioural disorders due to use of other stimulants, including caffeine, unspecified mental and behavioural disorder, methylamphetamine |
| F1592 | Mental and behavioural disorders due to use of other stimulants, including caffeine, unspecified mental and behavioural disorder, methylenedioxy methamphetamine |
| F1599 | Mental and behavioural disorders due to use of other stimulants, including caffeine, unspecified mental and behavioural disorder, other specified stimulants |
| F1601 | Mental and behavioural disorders due to use of hallucinogens, acute intoxication, ketamine |
| F161 | Mental and behavioural disorders due to use of hallucinogens, harmful use |
| F1610 | Mental and behavioural disorders due to use of hallucinogens, harmful use, unspecified hallucinogen |
| F1611 | Mental and behavioural disorders due to use of hallucinogens, harmful use, ketamine |
| F1619 | Mental and behavioural disorders due to use of hallucinogens, harmful use, other specified hallucinogen |
| F162 | Mental and behavioural disorders due to use of hallucinogens, dependence syndrome |
| F165 | Mental and behavioural disorders due to use of hallucinogens, psychotic disorder |
| F169 | Mental and behavioural disorders due to use of hallucinogens, unspecified mental and behavioural disorder |
| F1690 | Mental and behavioural disorders due to use of hallucinogens, unspecified mental and behavioural disorder, unspecified hallucinogen |
| F181 | Mental and behavioural disorders due to use of volatile solvents, harmful use |
| F182 | Mental and behavioural disorders due to use of volatile solvents, dependence syndrome |
| F188 | Mental and behavioural disorders due to use of volatile solvents, other mental and behavioural disorders |
| F189 | Mental and behavioural disorders due to use of volatile solvents, unspecified mental and behavioural disorder |
| F190 | Mental and behavioural disorders due to multiple drug use and use of psychoactive substances, acute intoxication |
| F191 | Mental and behavioural disorders due to multiple drug use and use of psychoactive substances, harmful use |
| F192 | Mental and behavioural disorders due to multiple drug use and use of psychoactive substances, dependence syndrome |
| F193 | Mental and behavioural disorders due to multiple drug use and use of psychoactive substances, withdrawal state |
| F195 | Mental and behavioural disorders due to multiple drug use and use of psychoactive substances, psychotic disorder |
| F198 | Mental and behavioural disorders due to multiple drug use and use of psychoactive substances, other mental and behavioural disorders |
| F199 | Mental and behavioural disorders due to multiple drug use and use of psychoactive substances, unspecified mental and behavioural disorder |
| G251 | Drug-induced tremor |
| I427 | Cardiomyopathy due to drugs and other external agents |
| I952 | Hypotension due to drugs |
| O355 | Maternal care for (suspected) damage to fetus by drugs |
| N141 | Nephropathy induced by other drugs, medicaments and biological substances |
| Z715 | Counselling and surveillance for drug use disorder |
| Z722 | Drug use |
| Z8642 | Personal history of drug use disorder |
| P044 | Fetus and newborn affected by maternal use of drugs of addiction |

**Supplementary Table 2: Association of interaction between planned birth, birthweight categories and parity with study outcomes^*^ including those that underwent spontaneous labour between 39^+0^ and 39^+6^ in the expectant management cohort**

| **Perinatal Mortality** | **OR (95% CI)** | **p-value** |
| --- | --- | --- |
| Planned Birth and Parity |  |  |
| Expectant Management#Nulliparous | 1.36 (1.06, 1.73) | 0.01 |
| Planned Birth#Nulliparous | 0.49 (0.17, 1.42) | 0.19 |
|  |  |  |
| Planned Birth and Birthweight Category |  |  |
| Expectant Management#SGA <10^th^ | 2.81 (2.07, 3.82) | <0.001 |
| Expectant Management#LGA >90^th^ | 0.78 (0.48, 1.24) | 0.29 |
| Planned Birth#SGA <10^th^ | 1.77 (0.53, 5.97) | 0.36 |
| Planned Birth#LGA >90^th^ | 0.32 (0.04, 2.37) | 0.26 |
|  |  |  |
| Method of Planned Birth and Parity |  |  |
| Expectant Management#Nulliparous | 1.36 (1.06, 1.73) | 0.01 |
| Induction of Labour#Nulliparous | 0.29 (0.06, 1.33) | 0.11 |
| Scheduled Caesarean#Nulliparous | 0.74 (0.16, 3.39) | 0.70 |
|  |  |  |
| Method of Planned Birth and Birthweight Category |  |  |
| Expectant Management#SGA <10^th^ | 2.81 (2.07, 3.82) | <0.001 |
| Expectant Management#LGA >90^th^ | 0.78 (0.48, 1.24) | 0.29 |
| Induction of Labour#SGA <10^th^ | 0.91 (0.12, 7.12) | 0.93 |
| Induction of Labour#LGA >90^th^ | 0.67 (0.09, 5.22) | 0.70 |
| Scheduled Caesarean#SGA <10^th^ | 2.96 (0.65, 13.5) | 0.16 |
| Scheduled Caesarean#LGA >90^th^ | # | # |
|  |  |  |
| **Antepartum Stillbirth** | **OR (95% CI)** | **p-value** |
| Planned Birth and Parity |  |  |
| Expectant Management#Nulliparous | 1.08 (0.71, 1.66) | 0.71 |
| Planned Birth#Nulliparous | 0.41 (0.05, 3.37) | 0.40 |
|  |  |  |
| Planned Birth and Birthweight Category |  |  |
| Expectant Management#SGA <10^th^ | 3.60 (2.21, 5.86) | <0.001 |
| Expectant Management#LGA >90^th^ | 0.50 (0.18, 1.38) | 0.18 |
| Planned Birth#SGA <10^th^ | 2.36 (0.28, 20.24) | 0.43 |
| Planned Birth#LGA >90^th^ | 1.27 (0.15, 10.91) | 0.83 |
|  |  |  |
| Method of Planned Birth and Parity |  |  |
| Expectant Management#Nulliparous | 1.08 (0.71, 1.66) | 0.71 |
| Induction of Labour#Nulliparous | 1.46 (0.09, 23.39) | 0.79 |
| Scheduled Caesarean#Nulliparous | # | # |
|  |  |  |
| Method of Planned Birth Birthweight Category |  |  |
| Expectant Management#SGA <10^th^ | 3.60 (2.21, 5.86) | <0.001 |
| Expectant Management#LGA >90^th^ | 0.50 (0.18, 1.38) | 0.18 |
| Induction of Labour #SGA <10^th^ | # | # |
| Induction of Labour#LGA >90^th^ | 6.68 (0.42, 106.79) | 0.18 |
| Scheduled Caesarean#SGA <10^th^ | 3.70 (0.41, 33.07) | 0.24 |
| Scheduled Caesarean# LGA >90^th^ | # | # |
|  |  |  |
| **Intrapartum Stillbirth** | **OR (95% CI)** | **p-value** |
| Planned Birth and Parity |  |  |
| Expectant Management#Nulliparous | 1.67 (1.04, 2.68) | 0.03 |
| Planned Birth#Nulliparous | # | # |
|  |  |  |
| Planned Birth and Birthweight Category |  |  |
| Expectant Management#SGA <10^th^ | 2.83 (1.56, 5.11) | 0.001 |
| Expectant Management#LGA >90^th^ | 0.94 (0.40, 2.20) | 0.89 |
| Planned Birth#SGA <10^th^ | # | # |
| Planned Birth#LGA >90^th^ | # | # |
|  |  |  |
| Method of Planned Birth and Parity |  |  |
| Expectant Management#Nulliparous | 1.67 (1.04, 2.68) | 0.03 |
| Induction of Labour#Nulliparous | # | # |
| Scheduled Caesarean#Nulliparous | # | # |
|  |  |  |
| Method of Planned Birth and Birthweight Category |  |  |
| Expectant Management#SGA <10^th^ | 2.83 (1.56, 5.11) | 0.001 |
| Expectant Management#LGA >90^th^ | 0.94 (0.40, 2.20) | 0.89 |
| Induction of Labour#SGA <10^th^ | # | # |
| Induction of Labour#LGA >90^th^ | # | # |
| Scheduled Caesarean#SGA <10^th^ | # | # |
| Scheduled Caesarean# LGA >90^th^ | # | # |
|  |  |  |
| **Neonatal Death** | **OR (95% CI)** | **p-value** |
| Planned Birth and Parity |  |  |
| Expectant Management#Nulliparous | 1.59 (1.07, 2.35) | 0.02 |
| Planned Birth 39+0 – 39+6#Nulliparous | 0.64 (0.18, 2.26) | 0.49 |
|  |  |  |
| Planned Birth and Birthweight Category |  |  |
| Expectant Management#SGA <10^th^ | 2.29 (1.35, 3.87) | 0.002 |
| Expectant Management#LGA >90^th^ | 0.94 (0.47, 1.88) | 0.87 |
| Planned Birth#SGA <10^th^ | 1.93 (0.44, 8.55) | 0.39 |
| Planned Birth#LGA >90^th^ | # | # |
|  |  |  |
| Method of Planned Birth and Parity |  |  |
| Expectant Management#Nulliparous | 1.59 (1.07, 2.35) | 0.02 |
| Induction of Labour#Nulliparous | 0.23 (0.03, 1.84) | 0.17 |
| Scheduled Caesarean#Nulliparous | 1.49 (0.29, 7.66) | 0.64 |
|  |  |  |
| Method of Planned Birth and Birthweight Category |  |  |
| Expectant Management#SGA <10^th^ | 2.29 (1.35, 3.87) | 0.002 |
| Expectant Management#LGA >90^th^ | 0.94 (0.47, 1.88) | 0.87 |
| Induction of Labour#SGA <10^th^ | 1.40 (0.17, 11.35) | 0.76 |
| Induction of Labour#LGA >90^th^ | # | # |
| Scheduled Caesarean#SGA <10^th^ | 2.56 (0.31, 21.23) | 0.39 |
| Scheduled Caesarean# LGA >90^th^ | # | # |
|  |  |  |
| **Severe Neurological Morbidity** | **OR (95% CI)** | **p-value** |
| Planned Birth and Parity |  |  |
| Expectant Management#Nulliparous | 2.05 (1.91, 2.20) | <0.001 |
| Planned Birth#Nulliparous | 2.27 (1.88, 2.74) | <0.001 |
|  |  |  |
| Planned Birth and Birthweight Category |  |  |
| Expectant Management#SGA <10^th^ | 1.58 (1.41, 1.76) | <0.001 |
| Expectant Management#LGA >90^th^ | 1.22 (1.09, 1.36) | <0.001 |
| Planned Birth#SGA <10^th^ | 1.65 (1.19, 2.27) | 0.002 |
| Planned Birth#LGA >90^th^ | 1.54 (1.19, 1.98) | 0.001 |
|  |  |  |
| Method of Planned Birth and Parity |  |  |
| Expectant Management#Nulliparous | 2.05 (1.91, 2.20) | <0.001 |
| Induction of Labour#Nulliparous | 2.05 (1.63, 2.60) | <0.001 |
| Scheduled Caesarean#Nulliparous | 1.51 (1.06, 2.15) | 0.02 |
|  |  |  |
| Method of Planned Birth and Birthweight Category |  |  |
| Expectant Management#SGA <10^th^ | 1.58 (1.41, 1.76) | <0.001 |
| Expectant Management#LGA >90^th^ | 1.22 (1.09, 1.36) | <0.001 |
| Induction of Labour#SGA <10^th^ | 1.53 (1.05, 2.23) | 0.03 |
| Induction of Labour#LGA >90^th^ | 1.75 (1.28, 2.38) | <0.001 |
| Scheduled Caesarean#SGA <10^th^ | 1.32 (0.69, 2.52) | 0.40 |
| Scheduled Caesarean#LGA >90^th^ | 1.28 (0.82, 2.01) | 0.28 |
|  |  |  |
| **Severe Non-Neurological Morbidity** | **OR (95% CI)** | **p-value** |
| Planned Birth and Parity |  |  |
| Expectant Management#Nulliparous | 2.36 (2.30, 2.42) | <0.001 |
| Planned Birth#Nulliparous | 2.22 (2.09, 2.35) | <0.001 |
|  |  |  |
| Planned Birth and Birthweight Category |  |  |
| Expectant Management#SGA <10^th^ | 1.39 (1.33, 1.45) | <0.001 |
| Expectant Management#LGA >90^th^ | 1.62 (1.57, 1.68) | <0.001 |
| Planned Birth#SGA <10^th^ | 2.41 (2.21, 2.64) | <0.001 |
| Planned Birth#LGA >90^th^ | 2.05 (1.90, 2.21) | <0.001 |
|  |  |  |
| Method of Planned Birth and Parity |  |  |
| Expectant Management#Nulliparous | 2.36 (2.30, 2.42) | <0.001 |
| Induction of Labour#Nulliparous | 2.76 (2.55, 2.98) | <0.001 |
| Scheduled Caesarean#Nulliparous | 0.98 (0.88, 1.10) | 0.78 |
|  |  |  |
| Method of Planned Birth and Birthweight Category |  |  |
| Expectant Management#SGA <10^th^ | 1.39 (1.33, 1.45) | <0.001 |
| Expectant Management#LGA >90^th^ | 1.62 (1.57, 1.68) | <0.001 |
| Induction of Labour#SGA <10^th^ | 2.09 (1.87, 2.34) | <0.001 |
| Induction of Labour#LGA >90^th^ | 1.64 (1.48, 1.83) | <0.001 |
| Scheduled Caesarean#SGA <10^th^ | 2.40 (2.05, 2.81) | <0.001 |
| Scheduled Caesarean# LGA >90^th^ | 2.76 (2.49, 3.07) | <0.001 |
|  |  |  |
| **Severe Maternal Outcome** | **OR (95% CI)** | **p-value** |
| Planned Birth and Parity |  |  |
| Expectant Management#Nulliparous | 1.66 (1.63, 1.70) | <0.001 |
| Planned Birth#Nulliparous | 1.73 (1.65, 1.81) | <0.001 |
|  |  |  |
| Planned Birth and Birthweight Category |  |  |
| Expectant Management#SGA <10^th^ | 0.85 (0.82, 0.89) | <0.001 |
| Expectant Management#LGA >90^th^ | 1.50 (1.45, 1.54) | <0.001 |
| Planned Birth 39+0 – 39+6#SGA <10^th^ | 1.01 (0.93, 1.10) | 0.824 |
| Planned Birth 39+0 – 39+6#LGA >90^th^ | 1.30 (1.22, 1.38) | <0.001 |
|  |  |  |
| Method of Planned Birth and Parity |  |  |
| Expectant Management#Nulliparous | 1.66 (1.63, 1.7) | <0.001 |
| Induction of Labour#Nulliparous | 1.82 (1.70, 1.94) | <0.001 |
| Scheduled Caesarean#Nulliparous | 1.60 (1.51, 1.71) | <0.001 |
|  |  |  |
| Method of Planned Birth and Birthweight Category |  |  |
| Expectant Management#SGA <10^th^ | 0.85 (0.82,0.89) | <0.001 |
| Expectant Management#LGA >90^th^ | 1.50 (1.45, 1.54) | <0.001 |
| Induction of Labour#SGA <10^th^ | 0.98 (0.87,1.10) | 0.69 |
| Induction of Labour#LGA >90^th^ | 1.50 (1.37, 1.64) | <0.001 |
| Scheduled Caesarean#SGA <10^th^ | 1.01(0.89,1.15) | 0.84 |
| Scheduled Caesarean# LGA >90^th^ | 1.17 (1.08, 1.27) | <0.001 |
|  |  |  |
| **Maternal-Infant Separation** | **OR (95% CI)** | **p-value** |
| Planned Birth and Parity |  |  |
| Expectant Management#Nulliparous | 2.15 (2.10, 2.21) | <0.001 |
| Planned Birth#Nulliparous | 1.58 (1.51, 1.66) | <0.001 |
|  |  |  |
| Planned Birth and Birthweight Category |  |  |
| Expectant Management#SGA <10^th^ | 1.55 (1.50, 1.61) | <0.001 |
| Expectant Management#LGA >90^th^ | 1.59 (1.54, 1.65) | <0.001 |
| Planned Birth#SGA <10^th^ | 2.29 (2.12, 2.47) | <0.001 |
| Planned Birth#LGA >90^th^ | 1.66 (1.55, 1.77) | <0.001 |
|  |  |  |
| Method of Planned Birth and Parity |  |  |
| Expectant Management#Nulliparous | 2.15 (2.10, 2.21) | <0.001 |
| Induction of Labour#Nulliparous | 2.35 (2.18, 2.52) | <0.001 |
| Scheduled Caesarean#Nulliparous | 0.92 (0.85, 1.00) | 0.04 |
|  |  |  |
| Method of Planned Birth and Birthweight Category |  |  |
| Expectant Management#SGA <10^th^ | 1.55 (1.50, 1.61) | <0.001 |
| Expectant Management#LGA >90^th^ | 1.59 (1.54, 1.65) | <0.001 |
| Induction of Labour#SGA <10^th^ | 2.42 (2.19, 2.67) | <0.001 |
| Induction of Labour#LGA >90^th^ | 1.49 (1.34, 1.64) | <0.001 |
| Scheduled Caesarean#SGA <10^th^ | 1.98 (1.76, 2.23) | <0.001 |
| Scheduled Caesarean# LGA >90^th^ | 1.80 (1.66, 1.96) | <0.001 |
|  |  |  |
| **Severe Perineal Trauma** | **OR (95% CI)** | **p-value** |
| Planned Birth and Parity |  |  |
| Expectant Management#Nulliparous | 3.71 (3.54, 3.90) | <0.001 |
| Induction of Labour#Nulliparous | 4.24 (3.53, 5.09) | <0.001 |
|  |  |  |
| Planned Birth and Birthweight Category |  |  |
| Expectant Management#SGA <10^th^ | 0.66 (0.60, 0.73) | <0.001 |
| Expectant Management#LGA >90^th^ | 1.32 (1.25, 1.41) | <0.001 |
| Induction of Labour#SGA <10^th^ | 0.62 (0.43, 0.89) | 0.009 |
| Induction of Labour#LGA >90^th^ | 1.47 (1.18, 1.82) | 0.001 |
|  |  |  |
| **Shoulder Dystocia** | **OR (95% CI)** | **p-value** |
| Planned Birth and Parity |  |  |
| Expectant Management#Nulliparous | 0.55 (0.52, 0.59) | <0.001 |
| Induction of Labour#Nulliparous | 0.72 (0.61, 0.86) | <0.001 |
|  |  |  |
| Planned Birth and Birthweight Category |  |  |
| Expectant Management#SGA <10^th^ | 0.09 (0.07, 0.13) | <0.001 |
| Expectant Management#LGA >90^th^ | 5.86 (5.55, 6.18) | <0.001 |
| Induction of Labour#SGA <10^th^ | 0.03 (0.00, 0.21) | <0.001 |
| Induction of Labour#LGA >90^th^ | 5.55 (4.69, 6.58) | <0.001 |
|  |  |  |
| **Caesarean Birth**** | **OR (95% CI)** | **p-value** |
| Planned Birth and Parity |  |  |
| Expectant Management#Nulliparous | 3.55 (3.48, 3.61) | <0.001 |
| Induction of Labour#Nulliparous | 7.30 (6.84, 7.78) | <0.001 |
|  |  |  |
| Planned Birth and Birthweight Category |  |  |
| Expectant Management#SGA <10^th^ | 1.08 (1.04, 1.11) | <0.001 |
| Expectant Management#LGA >90^th^ | 1.46 (1.42, 1.5) | <0.001 |
| Induction of Labour#SGA <10^th^ | 1.12 (1.02, 1.23) | 0.02 |
| Induction of Labour#LGA >90^th^ | 1.35 (1.25, 1.46) | <0.001 |

# no observations; BW Birthweight

**^*^**By interaction we mean modification (by birthweight centile or parity) of the effect of the exposure (planned birth) on specific study outcomes. Interactions were deemed significant at an alpha of 0.05.

** Caesarean birth after attempted vaginal delivery

**Supplementary Table 3: Multivariable logistic regression analyses of the association of planned birth at 39^+0^ to 39^+6^ weeks with perinatal mortality, severe neurological morbidity and severe non-neurological morbidity for all births, Small for Gestational Age, Large for Gestational Age, Nulliparous and Multiparous cohorts**

|  | **Perinatal Mortality** | | | | **Severe Neurological Morbidity** | | | | **Severe Non-Neurological Morbidity** | | | |
| --- | --- | --- | --- | --- | --- | --- | --- | --- | --- | --- | --- | --- |
|  | **OR**  **(95% CI)** | **p-value** | **aOR**  **(95% CI)** | **p-value** | **OR**  **(95% CI)** | **p-value** | **aOR**  **(95% CI)** | **p-value** | **OR**  **(95% CI)** | **p-value** | **aOR**  **(95% CI)** | **p-value** |
| **All Planned Births** | 0.36 (0.24, 0.55) | <0.0001 | 0.48 (0.30, 0.76)^a^ | 0.002 | 0.52 (0.47, 0.58) | <0.0001 | 0.46 (0.39, 0.53)^d^ | <0.0001 | 0.73 (0.71, 0.76) | <0.0001 | 0.65 (0.62, 0.68)^i^ | <0.0001 |
| **SGA Births** | 0.26 (0.08, 0.84) | 0.03 | 0.31 (0.10, 1.01)^b^ | 0.05 | 0.69 (0.42, 1.14) | 0.15 | 0.58 (0.35, 0.95)^e^ | 0.03 | 1.14 (1.04, 1.26) | 0.004 | 1.04 (0.89, 1.20)^j^ | 0.63 |
| **LGA Births** | 0.17 (0.02, 1.28) | 0.09 | 0.18 (0.02, 1.31)^c^ | 0.09 | 0.64 (0.50, 0.82) | 0.0005 | 0.54 (0.39, 0.74)^f^ | 0.0002 | 0.83 (0.77, 0.89) | <0.0001 | 0.73 (0.67, 0.80)^j^ | <0.0001 |
| **Nulliparous** | NA | NA | NA | NA | 0.62 (0.54, 0.72) | <0.0001 | 0.53 (0.45, 0.63)^g^ | <0.0001 | 0.81 (0.78, 0.85) | <0.0001 | 0.59 (0.56, 0.63)^k^ | <0.0001 |
| **Multiparous** | NA | NA | NA | NA | 0.56 (0.49, 0.65) | <0.0001 | 0.44 (0.37, 0.52)^h^ | <0.0001 | 0.86 (0.82, 0.90) | <0.0001 | 0.67 (0.63, 0.71)^l^ | <0.0001 |

OR (95% CI) Odds Ratio and 95% Confidence Interval; SGA Small for Gestational Age; aOR Adjusted Odds Ratio; NA not applicable because interaction not significant.

^a^Adjusted for BMI, smoking, illicit drugs, previous stillbirth, year of birth and planned birth#birthweight centile; ^b^Adjusted for previous stillbirth, infant sex and year of birth; ^c^Adjusted for low SES; ^d^ Adjusted for maternal age, BMI, maternal country of birth, low SES, smoking, alcohol, illicit drugs, previous stillbirth, assisted conception, infant sex, year of birth, planned birth#birthweight centile and planned birth#parity; ^e^Adjusted for BMI, low SES, smoking, illicit drugs, infant sex, year of birth and planned birth#parity; ^f^Adjusted for maternal age, BMI, smoking, alcohol, infant sex, year of birth and planned birth#parity; ^g^Adjusted for maternal age, BMI, low SES, smoking, illicit drugs, assisted conception, infant sex, year of birth and planned birth#birthweight centile; ^h^Adjusted for BMI, low SES, smoking, alcohol, illicit drugs, previous stillbirth, infant sex, year of birth and planned birth#birthweight centile; ^i^Adjusted for BMI, maternal country of birth, smoking, illicit drugs, assisted conception, infant sex, year of birth, planned birth#parity and planned birth#birthweight centile; ^j^ Adjusted for BMI, maternal country of birth, smoking, illicit drugs, assisted conception, infant sex, year of birth and planned birth#parity; ^k^Adjusted for BMI, maternal country of birth, smoking, illicit drugs, assisted conception, infant sex, year of birth and planned birth#birthweight centile; ^l^Adjusted for BMI, maternal country of birth, smoking, illicit drugs, infant sex, year of birth and planned birth#birthweight centile.

**Supplementary Table 4:** **Multivariable logistic regression analyses of the effect of planned birth at 39^+0^ to 39^+6^ weeks on Stillbirth, Neonatal Death, Maternal-Infant Separation, Severe Maternal Outcome for Small for Gestational Age, Large for Gestational Age, Nulliparous and Multiparous cohorts.**

|  | **Antepartum Stillbirth** | | | | **Intrapartum Stillbirth** | | | | **Neonatal Death** | | | | **Severe Maternal Outcome** | | | | **Maternal-Infant Separation** | | | |
| --- | --- | --- | --- | --- | --- | --- | --- | --- | --- | --- | --- | --- | --- | --- | --- | --- | --- | --- | --- | --- |
|  | **OR**  **(95% CI)** | **p-value** | **aOR**  **(95% CI)** | **p-value** | **OR**  **(95% CI)** | **p-value** | **aOR**  **(95% CI)** | **p-value** | **OR**  **(95% CI)** | **p-value** | **aOR**  **(95% CI)** | **p-value** | **OR**  **(95% CI)** | **p-value** | **aOR**  **(95% CI** | **p-value** | **OR**  **(95% CI)** | **p-value** | **aOR**  **(95% CI)** | **p-value** |
| **All Planned Births** | 0.31  (0.14, 0.68) | 0.003 | 0.38  0.15,0.97)^a^ | 0.04 | 0.11  (0.03, 0.44) | 0.002 | 0.16  (0.04, 0.66)^b^ | 0.01 | 0.56  (0.33, 0.96) | 0.04 | 0.74  (0.41, 1.35)^c^ | 0.33 | 0.89  (0.87, 0.91) | <0.0001 | 0.95  (0.92, 0.99)^f^ | 0.008 | 0.86  (0.84, 0.89) | <0.0001 | 1.04  (1.00, 1.08)^k^ | 0.08 |
| **SGA Births** | ## | ## | ## | ## | ## | ## | ## | ## | ## | ## | ## | ## | 1.06  (0.97, 1.16) | 0.22 | 1.08  (0.94, 1.24)^g^ | 0.30 | 1.21  (1.12, 1.31) | <0.0001 | 1.30  (1.15, 1.46)^f^ | <0.0001 |
| **LGA Births** | ## | ## | ## | ## | ## | ## | ## | ## | ## | ## | ## | ## | 0.78  (0.73, 0.82) | <0.001 | 0.83  (0.77, 0.89)^h^ | <0.001 | 0.85  (0.80, 0.91) | <0.0001 | 0.95  (0.87, 1.03)^l^ | 0.19 |
| **Nulliparous** | NA | NA | NA | NA | NA | NA | NA | NA | 0.31  (0.10, 1.00) | 0.05 | 0.35  (0.11, 1.11)^d^ | 0.08 | 0.99  (0.96, 1.03) | 0.69 | 0.97  (0.93, 1.01)^i^ | 0.12 | 0.81  (0.78, 0.85) | <0.0001 | 0.71  (0.67, 0.75)^m^ | <0.0001 |
| **Multiparous** | NA | NA | NA | NA | NA | NA | NA | NA | 0.78  (0.41, 1.47) | 0.44 | 0.94  (0.49, 1.82)^e^ | 0.86 | 0.96  (0.93, 0.99) | 0.004 | 0.95  (0.91, 0.98)^j^ | 0.003 | 1.11  (1.07, 1.15) | <0.0001 | 1.11  (1.07, 1.16)^n^ | <0.0001 |
| **Multiparous with no previous caesarean** |  |  |  |  |  |  |  |  |  |  |  |  |  |  |  |  | 1.11  (1.05, 1.16) | 0.0001 | 1.11  (1.04, 1.18)^n^ | 0.001 |

Ref Referent category; OR (95% CI) Odds Ratio and 95% Confidence Interval; SGA Small for Gestational Age; aOR Adjusted Odds Ratio; NA not applicable because interaction not significant; **## analyses not performed because insufficient failures (≤10) per variable.**^a^Adjusted for smoking, year of birth and interaction planned birth#birthweight centile; ^b^Adjusted for maternal age, previous stillbirth, year of birth and interaction planned birth#birthweight centile; ^c^Adjusted for drugs, year of birth and interaction planned birth#birthweight centile; ^d^Adjusted for year of birth; ^e^Adjusted for smoking, drugs and year of birth; ^f^Adjusted for BMI, maternal country of birth, smoking, illicit drugs, assisted conception, infant sex, year of birth and interaction planned birth#parity; ^g^Adjusted for maternal age, maternal country of birth low SES, illicit drugs, previous stillbirth, assisted conception, year of birth and interaction planned birth#parity; ^h^Adjusted for BMI, maternal country of birth, illicit drugs, assisted conception, year of birth and interaction planned birth#parity; ^i^Adjusted for maternal age, BMI, maternal country of birth, illicit drugs, assisted conception, year of birth and interaction planned birth#birthweight centile; ^j^Adjusted for maternal age, BMI, maternal country of birth, low SES, smoking, illicit drugs, previous stillbirth, assisted conception, year of birth and interaction planned birth#birthweight centile; ^k^Adjusted for maternal age, BMI, maternal country of birth, smoking, drugs, previous stillbirth, assisted conception, infant sex, year of birth and interactions planned birth#birthweight centile, planned birth#parity; ^l^Adjusted for maternal age, BMI, maternal country of birth, smoking, illicit drugs, assisted conception, infant sex, year of birth and interaction planned birth#parity; ^m^Adjusted for maternal age, BMI, maternal country of birth, smoking, illicit drugs, assisted conception, infant sex, year of birth and interaction planned birth#birthweight centile; ^n^Adjusted for maternal age, BMI, maternal country of birth, smoking, illicit drugs, previous stillbirth, assisted conception, infant sex, year of birth and interaction planned birth#birthweight centile.

**Supplementary Table 5: Multivariable logistic regression analyses of the effect of planned birth at 39^+0^ to 39^+6^ weeks on Caesarean Birth, Severe Perineal Trauma and Shoulder Dystocia for Small for Gestational Age, Large for Gestational Age, Nulliparous and Multiparous cohorts.**

|  | **Severe Perineal Trauma** | | | | **Shoulder Dystocia** | | | | **Caesarean Birth** | | | |
| --- | --- | --- | --- | --- | --- | --- | --- | --- | --- | --- | --- | --- |
|  | **OR**  **(95% CI)** | **p-value** | **aOR**  **(95% CI)** | **p-value** | **OR**  **(95% CI)** | **p-value** | **aOR**  **(95% CI)** | **p-value** | **OR**  **(95% CI)** | **p-value** | **aOR**  **(95% CI)** | **p-value** |
| **Induction of Labour** | 0.62 (0.57, 0.67) | <0.0001 | 0.53 (0.45, 0.63)^a^ | <0.0001 | 0.89 (0.82, 0.97) | 0.009 | 0.73 (0.64, 0.84)^f^ | <0.0001 | 0.90 (0.87, 0.92) | <0.0001 | 0.54 (0.51, 0.58)^j^ | <0.0001 |
| **SGA Births** | 0.57 (0.40, 0.81) | 0.002 | 0.45 (0.16, 1.24)^b^ | 0.12 | ## | ## | ## | ## | 0.94 (0.85, 1.03) | 0.19 | 0.63 (0.52, 0.76)^k^ | <0.0001 |
| **LGA Births** | 0.67 (0.55, 0.82) | <0.0001 | 0.69 (0.51, 0.93)^c^ | 0.02 | 0.83 (0.73, 0.95) | 0.007 | 0.77 (0.66, 0.90)^g^ | 0.001 | 0.83 (0.77, 0.90) | <0.0001 | 0.59 (0.52, 0.68)^l^ | <0.0001 |
| **Nulliparous** | 0.67 (0.61, 0.74) | <0.0001 | 0.63 (0.56, 0.70)^d^ | <0.0001 | 1.05 (0.90, 1.22) | 0.54 | 0.97 (0.81, 1.16)^h^ | 0.70 | 1.19 (1.15, 1.23) | <0.0001 | 1.12 (1.08, 1.17)^m^ | <0.0001 |
| **Multiparous** | 0.59 (0.50, 0.69) | <0.0001 | 0.46 (0.38, 0.56)^e^ | <0.0001 | 0.80 (0.72, 0.89) | <0.0001 | 0.71 (0.60, 0.82)^i^ | <0.0001 | 0.58 (0.55, 0.61) | <0.0001 | 0.53 (0.50, 0.57)^n^ | <0.0001 |
| **Multiparous with no previous caesarean** |  |  |  |  |  |  |  |  | 1.07 (1.00, 1.14) | 0.05 | 1.08 (0.99, 1.17)^n^ | 0.07 |

OR (95% CI) Odds Ratio and 95% Confidence Interval; SGA Small for Gestational Age; aOR Adjusted Odds Ratio.

^a^Adjusted for maternal age, maternal country of birth, smoking, illicit drugs, infant sex and year of birth, interactions planned birth#birthweight centile and planned birth#parity; ^b^Adjusted for maternal country of birth, smoking, infant sex and year of birth, interaction between planned birth#parity; ^c^Adjusted for maternal age, maternal country of birth, smoking, infant sex and year of birth, interaction between planned birth#parity; ^d^Adjusted for maternal age, BMI, maternal country of birth, low SES, smoking, illicit drugs, infant sex and year of birth, interaction planned birth#birthweight centile; ^e^Adjusted for maternal age, maternal country of birth, low SES, smoking, illicit drugs, infant sex and year of birth, interaction planned birth#birthweight centile; ^f^ Adjusted for maternal age, BMI, maternal country of birth, low SES, illicit drugs, infant sex and year of birth, interactions planned birth#birthweight centile and planned birth#parity; ^g^Adjusted for maternal age, infant sex and year of birth, interaction planned birth#parity; ^h^Adjusted for maternal age, low SES, illicit drugs, infant sex and year of birth, interaction planned birth#birthweight centile; ^i^Adjusted for maternal age, BMI, maternal country of birth, low SES, infant sex and year of birth, interaction planned birth#birthweight centile; ^j^Adjusted for maternal age, BMI, maternal country of birth, illicit drugs, previous stillbirth, assisted conception, infant sex and year of birth, interactions planned birth#birthweight centile and planned birth#parity; ^k^Adjusted for maternal age, BMI, maternal country of birth, smoking, assisted conception and infant sex, interaction planned birth#parity; ^l^Adjusted for maternal age, BMI, maternal country of birth, alcohol, assisted conception, infant sex and year of birth, interaction planned birth#parity; ^m^Adjusted for maternal age, BMI, maternal country of birth, assisted conception, infant sex and year of birth, interaction planned birth#birthweight centile; ^n^Adjusted for maternal age, BMI, maternal country of birth, low SES, illicit drugs, previous stillbirth, assisted conception, infant sex and year of birth, interaction planned birth#birthweight centile.
